# Structure and mechanism of monoclonal antibody binding to the junctional epitope of *Plasmodium falciparum* circumsporozoite protein

**DOI:** 10.1371/journal.ppat.1008373

**Published:** 2020-03-09

**Authors:** David Oyen, Jonathan L. Torres, Phillip C. Aoto, Yevel Flores-Garcia, Špela Binter, Tossapol Pholcharee, Sean Carroll, Sini Reponen, Rachael Wash, Qi Liang, Franck Lemiale, Emily Locke, Allan Bradley, C. Richter King, Daniel Emerling, Paul Kellam, Fidel Zavala, Andrew B. Ward, Ian A. Wilson

**Affiliations:** 1 Department of Integrative Structural and Computational Biology, The Scripps Research Institute, La Jolla, California, United States of America; 2 Department of Pharmacology, University of California at San Diego, La Jolla, California, United States of America; 3 Malaria Research Institute, Johns Hopkins Bloomberg School of Public Health, Baltimore, Maryland, United States of America; 4 Kymab Ltd., The Bennet Building (B930), Babraham Research Campus, Cambridge, United Kingdom; 5 Atreca Inc., South San Francisco, California, United States of America; 6 PATH’s Malaria Vaccine Initiative, PATH Center for Vaccine Innovation and Access, Washington, United States of America; 7 Wellcome Trust Sanger Institute, Hinxton, Cambridge, United Kingdom; 8 Department of Infectious Diseases, Faculty of Medicine, Imperial College London, London, United Kingdom; 9 The Skaggs Institute for Chemical Biology, The Scripps Research Institute, La Jolla, California, United States of America; National Institute of Allergy and Infectious Diseases, UNITED STATES

## Abstract

Lasting protection has long been a goal for malaria vaccines. The major surface antigen on *Plasmodium falciparum* sporozoites, the circumsporozoite protein (PfCSP), has been an attractive target for vaccine development and most protective antibodies studied to date interact with the central NANP repeat region of PfCSP. However, it remains unclear what structural and functional characteristics correlate with better protection by one antibody over another. Binding to the junctional region between the N-terminal domain and central NANP repeats has been proposed to result in superior protection: this region initiates with the only NPDP sequence followed immediately by NANP. Here, we isolated antibodies in Kymab mice immunized with full-length recombinant PfCSP and two protective antibodies were selected for further study with reactivity against the junctional region. X-ray and EM structures of two monoclonal antibodies, mAb667 and mAb668, shed light on their differential affinity and specificity for the junctional region. Importantly, these antibodies also bind to the NANP repeat region with equal or better affinity. A comparison with an NANP-only binding antibody (mAb317) revealed roughly similar but statistically distinct levels of protection against sporozoite challenge in mouse liver burden models, suggesting that junctional antibody protection might relate to the ability to also cross-react with the NANP repeat region. Our findings indicate that additional efforts are necessary to isolate a true junctional antibody with no or much reduced affinity to the NANP region to elucidate the role of the junctional epitope in protection.

## Author summary

The circumsporozoite protein (CSP) of *Plasmodium falciparum* malaria has been the foundation for the design of transmission blocking malaria vaccines. To date, the most promising CSP-based vaccine candidate is RTS,S, which consists of the central repeating NANP amino-acid sequence and the C-terminal domain of CSP fused to hepatitis B surface antigen that assembles into virus-like particles. Potential shortcomings of RTS,S includes the lack of other potential CSP epitopes such as the junctional epitope, which is located between the N-terminal domain of CSP and the start of the NANP repeat region. Here, we elicited antibodies against full-length CSP and screened for junctional epitope binding. We then used an array of biophysical techniques to elucidate the nature of the binding and tested the level of two protective antibodies in a mouse challenge model. Although the antibodies were able to bind both junctional and NANP epitopes, the in vivo data showed distinct levels of protection between themselves and also to an NANP-only binder. Our data suggest that their protection ability may be related to the strong cross-reactivity with NANP epitopes. Since all reported junctional antibodies to date have dual-specificity, we suggest that a true junctional binder with no or very low NANP affinity, if one can be found, is essential to evaluate the contribution of the junctional epitope to protection.

## Introduction

Malaria is a vector-borne parasitic disease that led to 435,000 deaths in 2017, mainly caused by infection with *Plasmodium falciparum* (Pf) [1]. Although malaria can be a treatable disease with effective detection and timely management, it remains a major public health threat [1]. One of the reasons that malaria is difficult to control is because of its complex life cycle in two host organisms. Female Anopheles mosquitoes transmit Pf to humans via sporozoites that are transferred during acquisition of a blood meal. Once deposited in the skin, sporozoites migrate into the blood stream and target the human liver, develop and appear in the blood as merozoites where they are responsible for the symptomatic stage of the life cycle, and eventually progress to the gametocyte sexual stage, which can be transmitted back to the mosquito. Vector control and case management have met with some success, but insecticide resistance is currently thwarting further progress [2]. Vaccine development on the other hand has focused on eliciting an immune response against *P*. *falciparum* sporozoites so as to prevent infection and thus transition to the symptomatic stage of malaria. Vaccine efforts against different targets from the erythrocytic stage of *P*. *falciparum* and also from gametocytes for transmission-blocking purpose are also being actively investigated [3]. Alternatively, delivery of antigens via attachment to liposomes is also being pursued for multivalent display of, for example, HIV-1 envelop trimers, where ease of production and ability to attach different antigens make these flexible platforms for antigen delivery, representing strategies yet to be explored for malaria [4–7]. Currently, the most advanced vaccine candidate is a recombinant protein based on the central NANP repeats and C-terminal region of the Pf circumsporozoite protein (GlaxoSmithKline RTS,S/AS01 vaccine) [8]. The RTS,S vaccine has completed Phase 3 clinical trials and support from the European Medicine Agency (EMA) and World Health Organization (WHO) has led to very recent implementation (April 23, 2019) of a large-scale pilot program for the vaccine in children under two in Malawi and shortly thereafter in Ghana and Kenya [9, 10]. Other approaches, such as radiation-attenuated, irradiated sporozoite vaccines (Sanaria PfSPZ vaccine), are in earlier stages of development [11]. PfCSP is GPI-anchored on the sporozoite surface and plays a critical role in sporozoite development, motility and hepatocyte invasion [12, 13]. Protective antibodies from vaccine candidates and from natural infection all target the central repeat region of PfCSP [14–18]. Typically, a single NPDP sequence starts the repeat region followed by NANP. Three of four NVDP repeats then alternate with NANP and a final NVDP is inserted in the middle of the NANP repeats, the number of which depends on the *P*. *falciparum* strain (e.g. 38 NANP repeats for the 3D7 strain) [19, 20]. RTS, S contains 19 NANP repeats and no NPDP or NVDP sequences [8]. Multiple copies of the antibodies are able to bind to the repeat region of a single PfCSP molecule, which suggests that cross-linking of B-cell receptors (BCR) may lead to a stronger BCR signal [21–25]. R21 is another form of RTS,S consisting of a single CSP-HBsAg fusion protein in a virus-like particle but with a greater density of CSP epitopes. R21 is reported to be more immunogenic and is in Phase1/2a clinical trials [26].

Recently, junctional antibodies have been isolated that are very effective in inhibiting infection in different mouse models [16, 17]. However, these antibodies appear to bind both to the NPDP (junctional epitope) and to the NANP repeats. Crystal structures have been determined for two of these antibodies, MGG4 and CIS43, in complex with the junctional peptide, but the mechanism of their elicitation that results in dual specificity remains poorly understood. Here, we report on crystal structures and negative-stain electron microscopy reconstructions for two highly protective antibodies, mAb667 and mAb668, which were obtained from immunization of Kymab transgenic mice containing human antibody genes with recombinant PfCSP [27], followed by antibody discovery using Atreca’s Immune Repertoire Capture technology. These antibodies display dual-specificity for the junctional peptide and the NANP repeats. We determined the contribution of individual amino acids to binding using mutational and molecular dynamics (MD) studies, providing further insights into the antibody specificity. Overall, these antibodies rely mainly on the first NANP repeat for junctional epitope binding, while allowing some promiscuous interactions with the flanking NPDP and NVDP repeats. Our data necessitate a reevaluation of the definition for a junctional epitope antibody; specifically, we propose that true junctional binders should be classified based on high specificity to NPDP or to the NVDP repeats and inability to bind to the NANP repeat region.

## Results

### Generation of mAb667 and mAb668

Kymab mice that are transgenic for the non-rearranged human antibody germline repertoire [27], were immunized with full-length recombinant CSP (rCSP) from Gennova Biopharmaceuticals Ltd (Pune, India) [28]. To determine whether the antibody responses were directed to native Pf antigens, sera from immunized mice were tested by an Indirect Immunofluorescence Assay (IFA) against whole Pf sporozoites. mAbs were derived from the immunized mice [27] using Atreca’s Immune Repertoire Capture (IRC) technology. The antibody repertoire was then further characterized by obtaining sequences of paired antibody genes from individual mice and analysis of the B-cell lineage sequences by IRC. A total of 2588 high quality antibody pairs were identified and assigned to clusters of putative lineages (S1 Fig). From each of the 37 lineages that received the highest scores, 48 selected antibody sequences were produced via gene synthesis and recombinant expression as fully human IgG1 antibodies and screened for binding to rCSP by ELISA. Of the nine ELISA-positive lineages (15 antibodies), antibodies from seven lineages bound to whole sporozoites by IFA, but only mAb667 and mAb668 demonstrated significant anti-malaria activity (S1 Table).

### Binding affinity of Fabs 667, 668 and 317 to the junctional epitope

**Fig 1:**
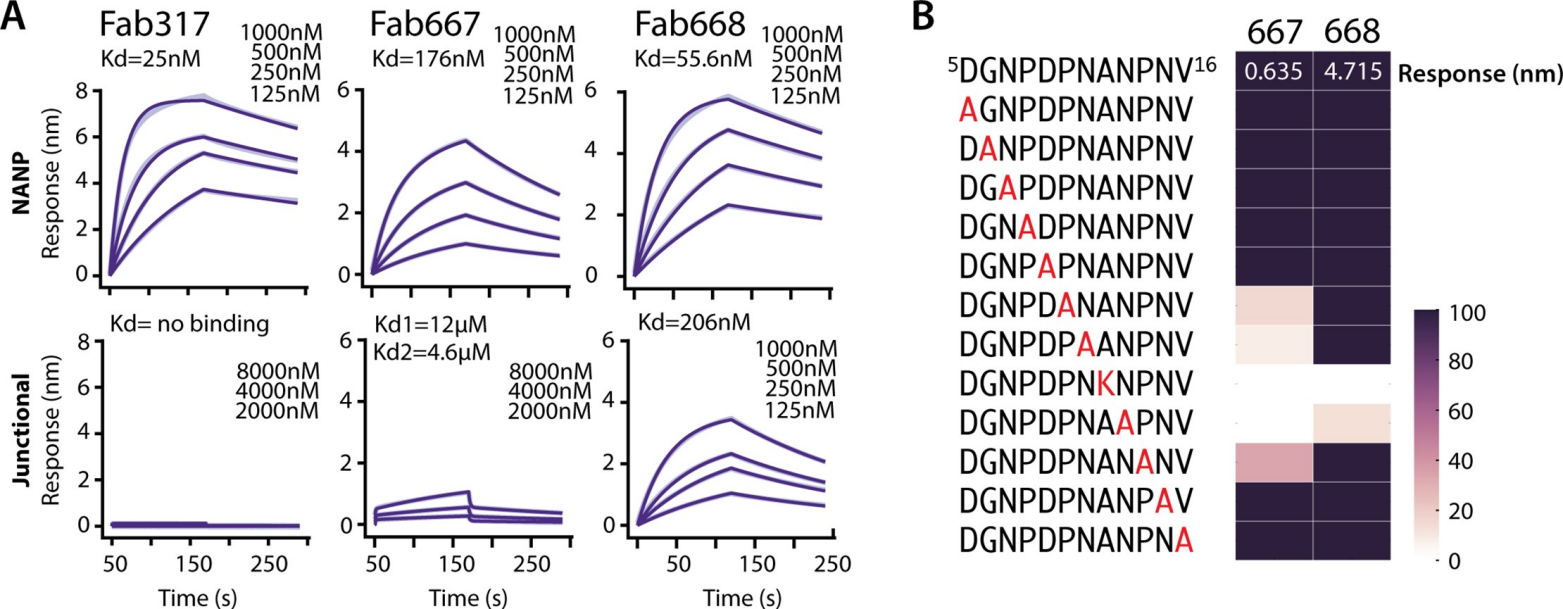
Binding studies to assess dual specificity for the junctional and NANP epitopes. (**A**). Biolayer Interferometry (BLI) was used to measure the kinetics of binding for Fab317, Fab667 and Fab668 to either the NPNANPNANPNANPNA (NANP) peptide or the KQPADGNPDPNANPNV (junctional) peptide. Binding curves are shown in light purple (wider lines) and fits are shown in dark purple (thinner lines). As the fits to the data are very good, the binding curves and fits overlap. (**B**) Alanine scan for the junctional peptide using BLI. The binding response (amplitude) is normalized to the wild-type junctional peptide. Binding is shown as a gradient from dark purple (native binding) to white (no binding).

Here, we analyzed the binding specificity of Fabs 667, 668 and 317 for junctional (KQPADGNPDPNANPNV) and NANP peptides (NPNA)_3_ using Biolayer Interferometry (BLI) (Fig 1A, S2 Table). Fab317 was isolated from a phase 2a RTS,S clinical trial and our previous affinity measurements indicated that it binds only NANP and not NVDP repeats, therefore, indicating that Fab317 is highly specific for NANP repeats [14]. All Fabs bound the NANP peptide with high affinity (K_d_’s from 25 to 170 nM), with Fab317 having the best binding as reflected by the lowest Kd. Only Fab668 bound the junctional peptide in the nM range, but with a K_d_ (206 nM) approximately 4-fold lower than to the NANP repeats (K_d_ = 56 nM) (Fig 1A, S2 Table). Fab667 displayed very weak binding to the junctional peptide. After inspection of the binding profiles and careful analysis of the residuals for each fitted model, Fab667 binding to the junctional peptide was best fit with a biphasic model (K_d1_ = 12 μM, K_d2_ = 4.6 μM). Regardless of the binding model, it is clear that Fab 668 binds the junctional peptide in the nanomolar range, Fab667 in the micromolar range, while Fab317 does not bind the junctional peptide at all.

To further define binding specificity, an alanine scan was performed on a slightly shorter junctional peptide ^5^DGNPDPNANPNV^16^ (n.b. we determined that ^1^KQPA^4^ was not involved in binding, see below) using BLI (Fig 1B). The low response unit (RU) of 0.37 for Fab667 compared to 4.72 for Fab668 at a single concentration confirmed the low affinity of Fab667 for the junctional peptide. Furthermore, this residual binding was completely dependent on the NANP repeat that is present within the junctional peptide, as mutation of any NANP residue to Ala (or to Lys for Ala substitution) abolished or substantially reduced binding. Only A12K and N13A mutations of those tested were disruptive to Fab668 binding, indicating that the NANP repeat was also important for Fab668 junctional peptide binding (Fig 1B).

### Crystal structures for Fab667 in complex with (NPNA)_3_ and Fab668 in complex with the junctional peptide (Junc)

**Fig 2:**
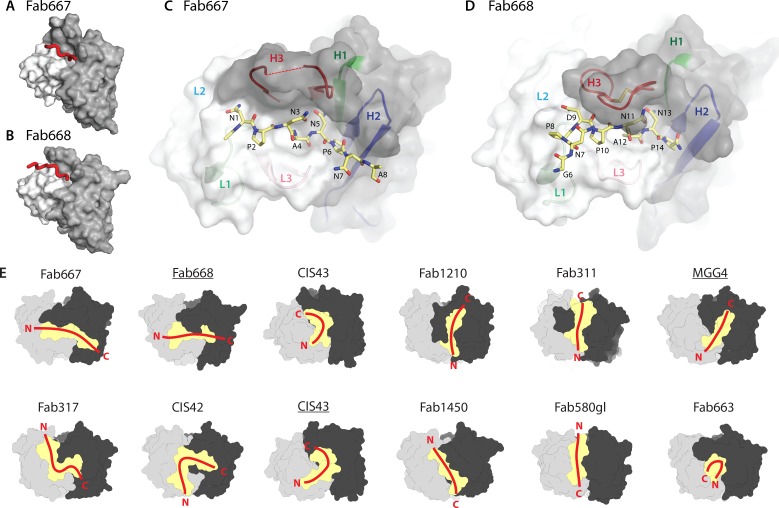
Structural analysis of binding of junctional and NANP repeat peptides to protective antibodies. Overview of the antibody paratopes for (**A**) Fab667 and (**B**) Fab668. The light and heavy chains are colored white and dark grey respectively. The peptide is shown as a red tube. Details of the paratopes and peptide conformation are shown for (**C**) Fab667 and (**D**) Fab668. Here the peptide is shown in the stick configuration (yellow carbons) and the residues are numbered. The CDR loops are colored according to their identity (H1: green, H2: blue, H3: red, L1: light green, L2: light blue and L3: pink). The tip of CDR H3 of Fab667 is disordered (^H^Gly^100C^) and is connected to the rest of CDR H3 by a dotted red line. (**E**) Comparison of Fab667 and Fab668 interactions with CSP peptides in previously published Fab structures (Fab317 (PDB code 6AXL), Fab311 (6AXK) [14], MGG4 (6BQB) [16], CIS43 (6B5O, 6B5L) and CIS42 (6B5T) [17], Fab1210 (6D01) and Fab1450 (6D11) [23], Fab580gl (6AZM) and Fab663 (5BK0) [15]). Paratopes are shown as a yellow surface with the heavy chain in black and light chain in grey. The overall conformations of each peptide and relative orientations are shown as red lines with their N- and C-termini indicated. Underlined Fab labels indicate structures in complex with junctional peptides.

Crystal structures were determined for Fab667 in complex with the (NPNA)_3_ peptide and for Fab668 in complex with the ^1^KQPADGNPDPNANP^14^ peptide to investigate the structural basis for junctional peptide binding (Fig 2A–2D, S3 Table). All peptides used for crystallization studies had N-terminal acetyl and C-terminal amide protection groups. Electron density for the first two NPNA repeats (Ac-NPNANPNA) are present in the Fab667 crystal structure (S2A Fig). In the Fab668 crystal structure, no electron density was found for the first five residues, but interpretable electron density was observed for NPDP and NANP as well as Gly^6^ (^6^GNPDPNANP^14^-NH_2_) (S2B Fig). Both Fabs bound the peptides in conformations and relative dispositions that are distinct from those observed in previous X-ray structures (Fig 2E). Instead of binding in a groove parallel to the heavy chain (HC)—light chain (LC) interface for many of these antibodies (Fabs1450, 580gl, 1210, 311) with some exceptions (Fabs 317, CIS42, CIS43), the peptides in complex with Fabs 667 and 668 are fully extended and bind perpendicular to the HC-LC interface (Fig 2E).

The buried surface area (BSA) for the Fab668-Junc complex is 516 Å^2^ on the Fab and 464 Å^2^ on the peptide. Similarly, for the Fab667-(NPNA)_3_ complex, the BSA is 501 Å^2^ on the Fab and 453 Å^2^ on the peptide. The overall conformation of the two peptides is identical for the central NANP repeat when bound to the two antibodies (S2C Fig), which is perhaps not surprising, since both antibodies originate from the same germline genes [VH1-3*01 and Vλ2–23*02, IgBlast [29]]. This analysis suggests that Fab668 binds to the NANP epitopes in a similar manner as Fab667. No type I β-turns were observed as in other antibody complexes with CSP repeat peptides [14, 15, 17, 23]; however, an Asn pseudo 3_10_ turn is present for the NAN sequence in the center of the peptide (residues 3–5 and 11–13 for the Fab667 and Fab668 complexes, respectively); the first Asn^i^ side chain hydrogen bonds with the backbone amide nitrogen of Asn^i+2^ in a similar hydrogen bond interaction, but in a different register, to the more commonly observed NPN-type Asn pseudo 3_10_ turn [14, 16, 24] (S2C Fig).

Approximately 80% of the total BSA on the peptide comes from only 6 amino acids (^1^NPNANP^6^) in the Fab667-(NPNA)_3_ crystal structure. Likewise, over 90% of the peptide BSA is contributed by only 7 residues (^8^PDPNANP^14^) in the Fab668-Junc structure (S3 Fig). Hence, the NANP repeat appears to be essential for binding for both antibodies, which is corroborated by the results from the alanine scan of the junctional peptide (Fig 1B). Further evidence for this conclusion is gained from the molecular interactions of the NANP repeat with Fab668, where Asn^13^ engages in three hydrogen bonds with ^H^Asn^52^ and the backbone amide of ^H^Gly^96^ and carbonyl of ^H^Cys^98^, while Asn^11^ makes two hydrogen bonds with the backbone carbonyls of ^H^Cys^98^ and ^H^Ser^100^ in addition to the intra-peptide hydrogen bond in the NAN-like pseudo 3_10_ turn (S4A Fig). Both asparagine residues flank Ala^12^, whose side chain protrudes into the paratope and makes van der Waals and CH/π interactions with ^H^Tyr^100G^ and ^L^Tyr^91^ (S4B Fig). Almost identical interactions are observed with Fab667 for ^3^NAN^5^ in the (NPNA)_3_ peptide, except for hydrogens bonds to the first Asn (S4C and S4D Fig).

Both Fab667 and Fab668 display slightly longer than average CDR H3 lengths (19 and 17 residues, respectively). However, CDR H3 of Fab667 is not completely ordered and no density is observed for ^H^Gly^100C^ at its tip (Fig 2C, dotted line). Fab668 contains a disulfide bond between ^H^Cys^98^ and ^H^Cys^100C^, which likely stabilizes the CDR H3 loop and its interaction with the peptide (Fig 2D). Fab668 CDR H3 is further stabilized by van der Waals interactions between ^H^Cys^98^, ^H^Tyr^100F^ and ^H^Tyr^100G^ (S4B Fig) that may in part explain the 3-fold higher affinity of Fab668.

### Molecular dynamics simulations show the importance of the NANP for binding

**Fig 3:**
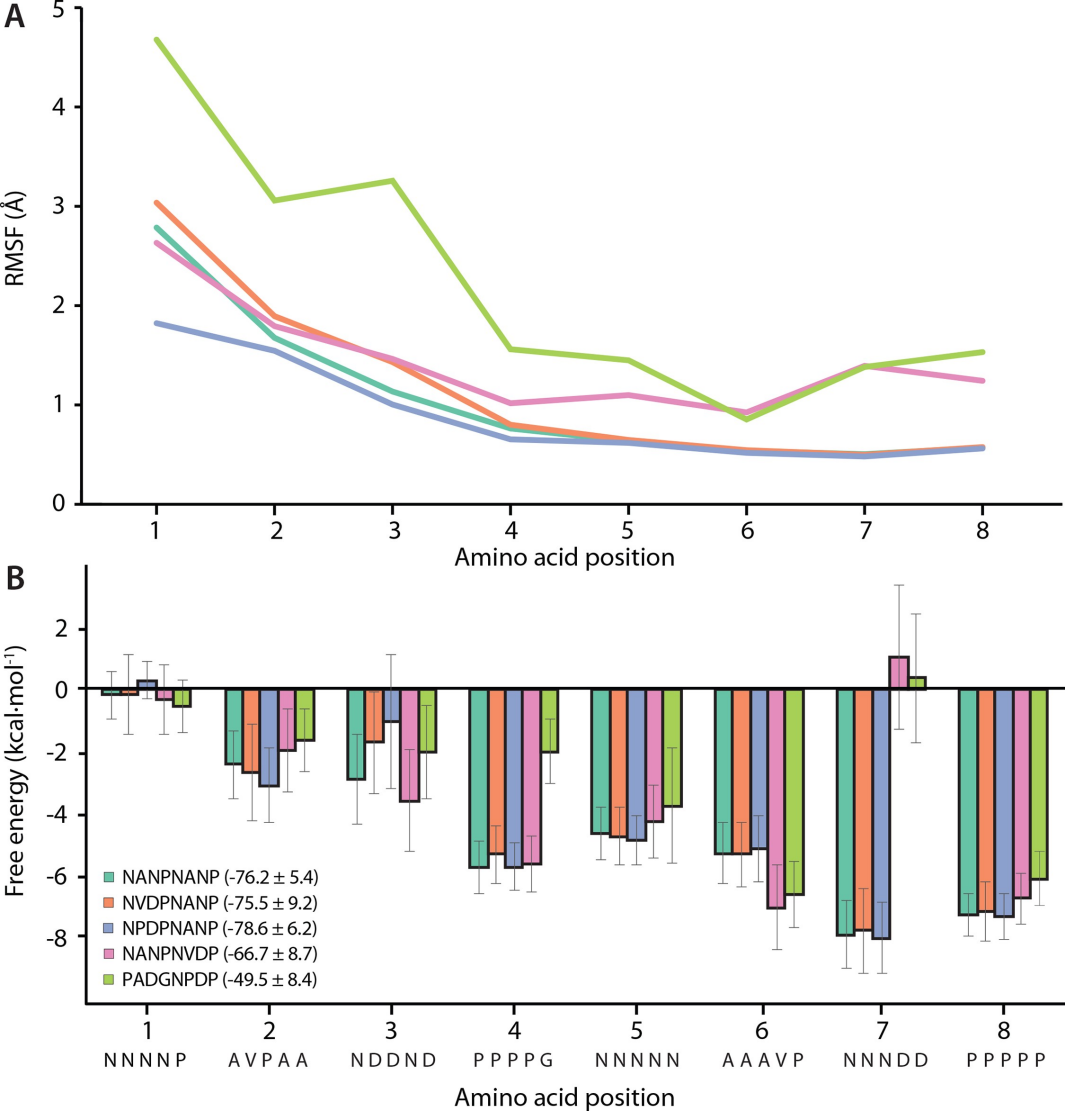
Molecular dynamics simulations to dissect the mechanism of Fab668 binding. Molecular dynamics (MD) simulations were undertaken to dissect binding to a variety of possible repeat epitopes for Fab668. **(A**) Root mean square fluctuations (RMSF) from 1.2 μs aggregate runs were calculated for each of the modeled peptide-Fab668 structures and plotted (NANPNANP: green, NVDPNANP: orange, NPDPNANP: blue, NANPNVDP: pink and PADGNPDP: lime, see also the graph legend in **B**). (**B**) Using the molecular mechanics / generalized Born solvent accessibility (MM/GBSA) approach, free energy contributions were calculated for each peptide and plotted as a function of amino-acid position (color code as in panel **A**).

To dissect the molecular mechanism for binding the junctional peptide, we performed in aggregate 1.5 μs of MD simulations per complex using the Fab668 crystal structure in which we modelled in five possible epitope registers of the junctional peptide containing one or two repeats that could potentially bind Fab668 [^1^NPDPNANP^8^, ^1^NANPNVDP^8^, ^1^NVDPNANP^8^ and ^1^NANPNANP^8^ (residues 1–4 and 5–8 for the first and second repeat, respectively), and ^1^PADGNPDP^8^ that includes the first repeat-like sequence (n.b. we did not perform such an analysis for Fab 667 as it does not bind the junctional peptide). These epitopes contain either pure NANP repeats, or NANP interspersed with NPDP or NVDP, and the last 4 amino acids of the N-terminal domain of PfCSP (PADG) linked to NPDP. NPDPNANP is the observed epitope in the crystal structure of the Fab668-junctional peptide complex and, as expected, has the lowest root mean square fluctuation (RMSF) calculated from MD simulations. This effect may also arise from the conformational restriction imposed on Asn^1^ by Pro^2^, which is not present in the other peptides. Interestingly, the ^1^NPDP^4^ repeat has a much higher RMSF compared to the ^5^NANP^8^ repeat within the same peptide (Fig 3A). Such a trend is also present in the other peptides (Fig 3A), which indicates that the binding of the first repeat may not contribute as much to the total binding as the binding of the second repeat. Fab668 prefers NANP for its second repeat, as substituting NANP for NVDP increases the RMSF for individual amino acids up to 1 Å each. Relative Gibbs free energy contributions to binding as estimated by the molecular mechanics / generalized Born solvent accessibility approach (MM/GBSA) yield further insights into the molecular mechanism of binding (Fig 3B). Considering these different peptide sequences, differences in amino-acid sequence occur at all positions except for position 5 (Asn^5^) and position 8 (Pro^8^). At position 1, Asn^1^ or Pro^1^ can both be accommodated, neither of which contributes to the overall free energy of binding. Positions 2 and 3 contribute more but less than positions 4 to 8, with Asn^3^ being slightly preferred more than Asp^3^. Position 4 prefers Pro^4^ since it has a 2.5-fold larger contribution to the overall free energy compared to Gly^4^ (-5 kcal/mol versus -2 kcal/mol), which indicates that Fab668 disfavors binding to the ^1^PADGNPDP^8^ sequence and likely needs two repeat-like sequences instead of the ^1^PADG^4^ sequence followed by NPDP at the immediate junction with the PfCSP N-terminal domain. This notion is also corroborated by the higher RMSF observed for this peptide and the weakest total Gibbs free energy for binding of -50 kcal/mol versus -76 kcal/mol for the NANPNANP peptide (Fig 3). A slightly larger contribution is observed for Val^6^ and Pro^6^ in position 6 as compared to the standard Ala^6^ in the NANP repeat, likely due to increased van der Waals or CH/π interactions. However, the most drastic change is observed in position 7 for substitution of Asn^7^ to Asp^7^. The latter does not contribute to favorable binding free energy, which is likely a manifestation of the negatively charged Asp^7^ that prefers an out-of-pocket orientation (S5A Fig), whereas the uncharged Asn^7^ is stabilized within the pocket by ND2 hydrogen bonds to ^H^Asn^52^ OD1 and ^H^Gly^96^ O (S5 Fig). Absence of this hydrogen bond network or the uncompensated negative charge of Asp^7^ then appears to contribute to weaker binding of NVDP relative to NANP.

We therefore investigated the role of protonation of Asp^7^ in NVDP binding. Although Asp^7^ should be deprotonated at neutral pH, if we protonate it in MD simulations (which could occur if completely buried in the antibody-antigen interface), binding stabilization similar to Asn^7^ is observed (S5 Fig). Therefore, constant-pH replica exchange MD (CpH-REMD), where discrete protonation states are sampled throughout the simulation, was employed to determine the pH-dependent distribution of NVDP conformations. Starting from an in-pocket conformation in which Asp^7^ is initially protonated, CpH-REMD was run with replica exchange in the pH dimension over pH 1 to 10, to enhance sampling, until convergence was observed in six independent simulations. The pKa of Asp^7^ was determined to be 6.1 from the CpH-REMD (S6 Fig). At physiological pH 7.4, the protonated NVDP conformation (~6%) contributes only weakly to binding. NVDP is therefore unlikely to bind to the NANP binding site on the Fab667 and Fab668 paratopes.

### Negative stain electron microscopy (nsEM) and multi-angle light scattering analysis for Fab667 and Fab668 in complex with rsCSP reveals binding stoichiometry

**Fig 4:**
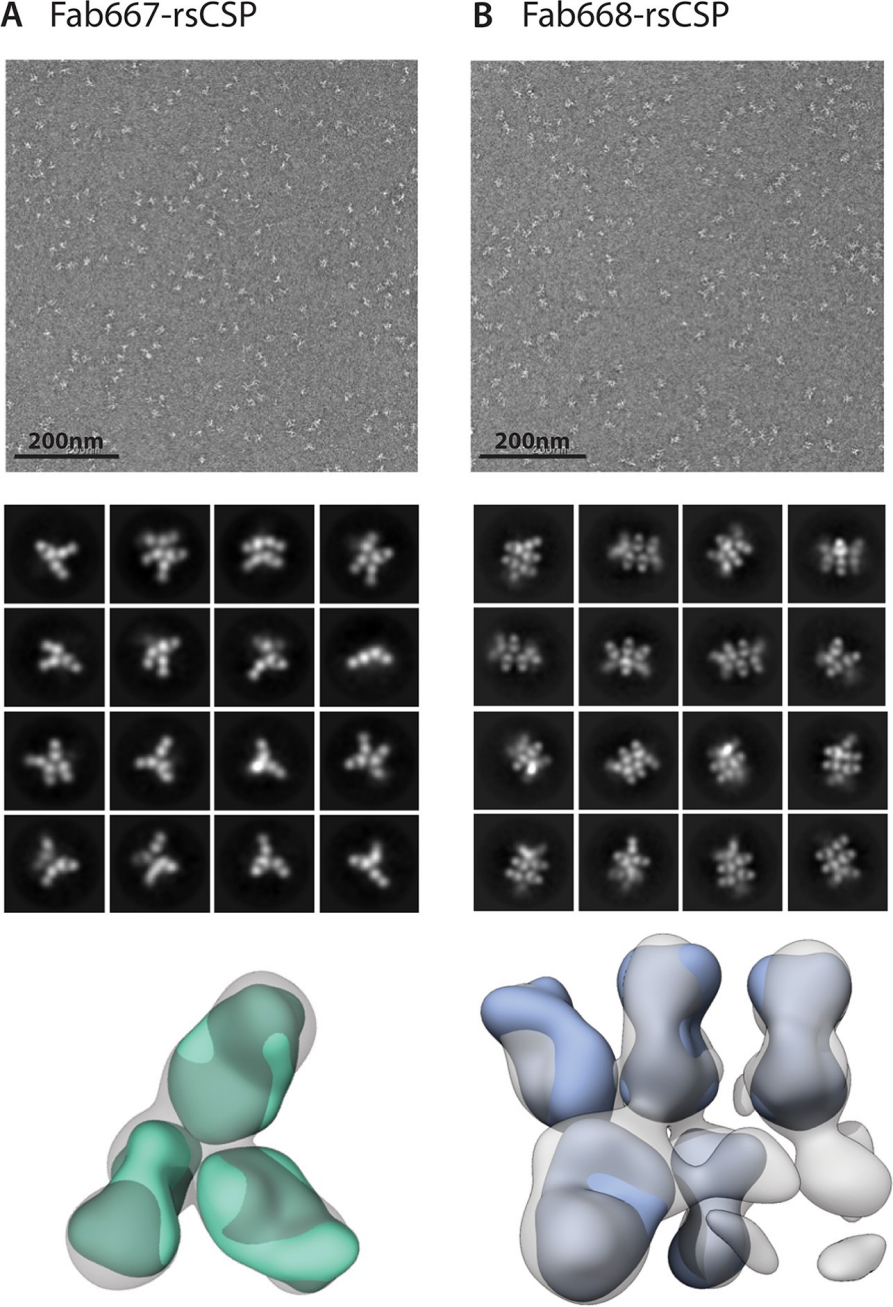
Negative-stain electron microscopy of rsCSP complexes. nsEM was used to assess the stoichiometry of the Fab667 and Fab668 complexes to rsCSP. 2D class averages and 3D reconstructions are shown for (**A**) Fab667-rsCSP and (**B**) Fab668-rsCSP complexes. Fab667-peptide (green) and Fab668 (cyan) were docked into their respective nsEM maps. The class averages and the 3D reconstructions both exhibit a lower apparent stoichiometry of Fabs bound in the Fab667 complex compared to the Fab668 complex.

Antibody binding to full-length CSP was approximated using a recombinant shortened construct (termed rsCSP, containing 19 NANP, 3 NVDP and 1 NPDP repeats), which has been shown to be a good mimic for CSP with its larger number of repeats (38 NANP, 4 NVDP and 1 NPDP repeats; 3D7 strain) [24]. Overall, reference-free 2D class averages were obtained with varying stoichiometries for both Fab667 and Fab668 in complex with rsCSP, which made 3D reconstructions difficult to converge (Fig 4, S7 Fig). However, the stoichiometry for the Fab667-rsCSP complex was on average lower (3 Fabs/complex) compared to the Fab668-rsCSP complex (5 Fabs/complex) that may reflect the lack of binding to the junctional region, which would reduce the number of available epitopes (Fig 4).

To further investigate these differences in stoichiometry, multi-angle light scattering was used to approximate the molecular mass of the two complexes (S8 Fig). The Fab667-rsCSP and Fab668-rsCSP complexes exhibited a total mass of about 290 ± 40 kDa and 480 ± 50 kDa, respectively. To calculate the number of Fabs per rsCSP, the molecular mass of rsCSP (33 kDa) was subtracted from the total mass and divided by the molecular mass of a Fab (50 kDa). On average, five Fab667 and nine Fab668 were bound to rsCSP, both of which were close to what was observed by nsEM. A possible explanation could be that some of the Fabs were eclipsed in the views observed in the 2D class averages or that the other regions are not ordered and thus unobserved. The former has been observed previously with the Fab311-rsCSP complex where we only observed 9 bound Fab311 by nsEM versus 11 by cryo-EM [14, 24]. Nonetheless, Fab668 had a higher stoichiometry of binding to rsCSP than Fab667.

### mAb667 and mAb668 inhibit sporozoite infection in vivo

**Fig 5:**
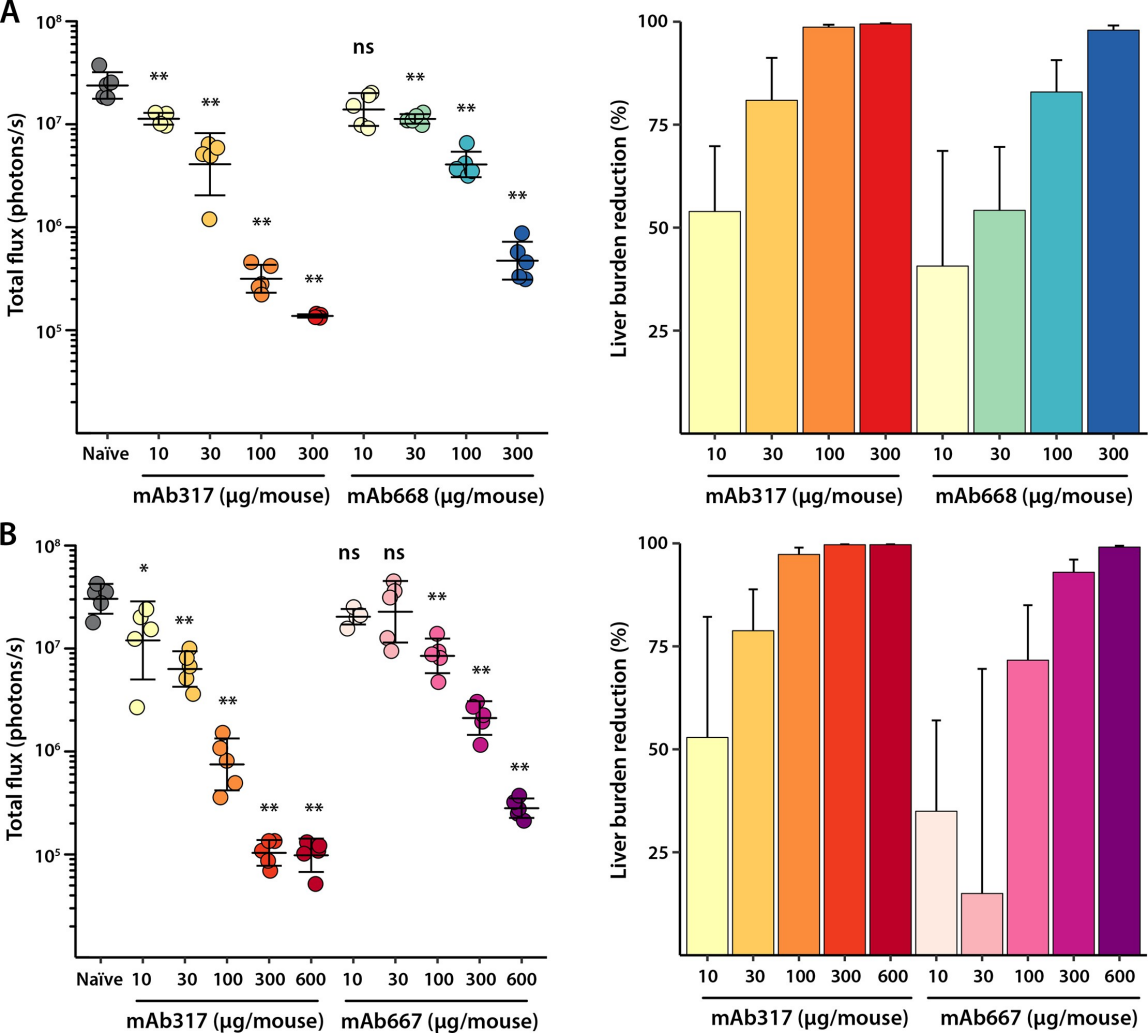
In vivo mice protection studies. Total flux (photons/second) of parasite liver burden load as measured by bioluminescence of luciferase-expressing transgenic *P*. *berghei* sporozoites after passive transfer of antibody in C57Bl/6 mice (5 mice per antibody concentration) and percent reduction of liver burden load as compared to naïve infected mice are plotted. (**A**) mAb317 (yellow to red colors) and mAb668 (yellow to blue colors) at four different antibody concentrations (300, 100, 30, 10 μg/mouse). (**B**) mAb317 (yellow to red colors) and mAb667 (pink to purple colors) at 5 different antibody concentrations (600, 300, 100, 30 and 10 μg/mouse). Mean and standard deviation are shown for the total flux of each mice group, while the plotted error was obtained by propagation of standard deviations for liver burden reduction. The Mann-Whitney U test was used to determine statistical significance compared to the naïve infected mice group (not significant (ns), * < 0.05 and ** < 0.01).

Reduction in liver-stage burden was then tested in a dose-dependent manner after injecting mice intravenously (IV) with monoclonal antibodies 667 (mAb667: 10 μg, 30 μg, 100 μg, 300 μg, 600 μg) and 668 (mAb668:10 μg, 30 μg, 100 μg, 300 μg) (Fig 5A and 5B). Five mice were used per mAb concentration and the mice were then challenged after 2 hours with 2x10^3^ chimeric sporozoites that stably express luciferase (*P*. *berghei* sporozoites expressing full-length *P*. *falciparum* CSP). Mice were injected with 100μl of D-luciferin 42 hours post-challenge and bioluminescence was measured using the IVIS spectrum. Both antibodies are potent, especially at high concentrations (300 and 600 μg/mouse) with >90% reduction in liver burden load as compared to naïve infected mice (Fig 5). mAb317 was used as a control for each experiment, since this anti-NANP antibody did not bind to the junctional region as shown by Biolayer Interferometry (BLI) (Fig 1). All antibodies are capable of strongly inhibiting liver stage development and exhibit only slight differences (although still statistically significant especially at higher doses) in their protective capacity (317 > 667 > 668) (Fig 5, S4 Table), which suggest that protection for mAb667 and mAb668 originates from their ability to also bind to the NANP repeat region.

## Discussion

The acquisition of structural information on the humoral immune response to sporozoite CSP is relatively new. Only recently have structures of anti-PfCSP antibodies been determined in complex with their respective epitopes [14–17, 23, 24]. These antibodies in general bind differently to the central region that includes the junctional and NANP repeats in PfCSP, and also display different levels of protection. Dual-specific binding to both the NANP epitope and the junctional epitope (NPDP repeat) has also been proposed to correlate with better protection [16, 17, 25]. Here, we studied two antibodies, mAb667 and mAb668, which have different propensities for binding the junctional epitope. Binding affinity studies with Fabs showed that only mAb668 binds well to the junctional region, while mAb667 is a very weak binder to that region. However, both antibodies bind well to the (NPNA)_3_ peptide. Alanine scan binding affinity studies and MD simulations further indicate that binding to the junctional peptide mostly relies on the NANP repeat that follows the only NPDP sequence in the junctional region of PfCSP. Thus, both mAb667 and mAb668 antibodies likely evolved mainly against the NANP repeat sequence. This notion agrees with the analysis done by Tan *et al*., where mutating the NANP repeat in a 19-mer junctional peptide (KQPADGNPDPNANPNVDPN) to AAAA abrogated binding for all antibodies tested (*20*). Further evidence comes from the Fab668-Junc crystal structure in which the NANP repeat has 2.5-fold more buried surface area (BSA) (268 Å^2^) compared to the NPDP repeat (102 Å^2^) within the same peptide. The NPDP binding pocket is shallow and promiscuous towards repeats with slightly varying sequence. It seems unlikely that these (and perhaps some other) antibodies have evolved specifically to bind *only* NPDP or NVDP repeats, given the small fraction that they represent within the abundance of NANP repeats: 2.3% for NPDP repeats and 9.3% for NVDP repeats (calculated using the number of repeat units present in the 3D7 strain of *P*. *falciparum*) and that they are always followed by an NANP repeat. Furthermore, we previously showed that mAb311 is also able to bind the junctional (NPDP) epitope and to the NVDP repeats [24], although this antibody was elicited by the RTS,S vaccine, which is devoid of NPDP or NVDP. However, it is notable that the RTS,S vaccine contains an MMAPDP sequence prior to the NANP repeats [8], which raises the question of whether this region might mimic the NPDP repeat. Whether junctional region binding is fortuitous or not, it is possible that binding close to Region 1 of the N-terminal domain may prevent the proteolytic cleavage of CSP that is necessary for hepatocyte invasion and, therefore, this characteristic may be advantageous for protection [17, 30]. However, our data suggest that some dual-specific antibodies are similarly or even less protective at higher doses as NANP-only antibodies, such as mAb317, in mice liver burden load experiments. Hence, eliciting dual-specific focused immune responses may not yield higher protection levels. Differentiating the contribution of the junctional epitope to protection has proved to be difficult, in part because we lack a mono-specific junctional antibody. Characteristics of such an optimal antibody would be high specificity against the NPDP and/or NVDP with little to no affinity to the NANP region. Immunizations with particles displaying the junctional peptide or with recombinant CSP lacking most of the NANP repeats may be required to obtain a true junctional binder. Future studies are essential to ascertain which attributes and epitopes are bona fide correlates of protection and, hence, which can aid in design of the most effective immunogens.

## Methods

### Mouse immunizations and sample collection

Kymab mice (a mix of 10-week old males and females) that are transgenic for the nonrearranged human antibody germline repertoire [27], were immunized three times at 3-week intervals using 20 μg of PfCSP formulated in Montanide ISA720 adjuvant, 30/70% v/v. Recombinant PfCSP (rCSP) was obtained from Gennova Biopharmaceuticals Ltd (Pune, India) and was produced as described previously [28]. The first two immunizations were administered IP using PfCSP formulated in Montanide ISA 720 adjuvant and the last immunization was performed IV with unadjuvanted CSP to allow for subsequent plasmablast collection. Sera were collected from whole blood on Days 0, 14, 28 and 49 (terminal bleed) and were used to monitor titers by ELISA. In addition to exsanguination, splenocytes were collected at time of sacrifice (Day 49). All immunizations, cell sorting and sequencing were done at Kymab Ltd. (UK).

### Splenocyte collection, plasmablast sorting, sequencing, and IRC

Single cell suspensions were prepared from the spleens of CSP-immunized mice and were sorted to identify plasmablasts/plasma single cells (CD138high, IgM^-^, IgD^-^, IgA^-^, CD3^-^). Atreca’s IRC technology was used to obtain full-length, natively paired, heavy and light chain variable region sequences from isolated plasmablasts. Cell lysis, reverse transcription, PCR, barcode assignment, sequence assembly, V(D)J assignment, and identification of mutations were performed as described previously [31, 32] with the following modifications: biotinylated Oligo(dT) was used for reverse transcription, cDNA was extracted using Streptavidin C1 beads (Life Technologies), PCR primers against mouse gamma, kappa, and lambda constant region sequences replaced human constant region primers, DNA concentrations were determined using qPCR (KAPA SYBR FAST qPCR Kit, Kapa Biosystems), and a minimum coverage of ten reads was required from each chain assembly to be included in the sequence repertoires. V(D)J assignment and mutation identification was performed using an implementation of SoDA [33].

Paired heavy and light chain sequences within each CSP-immunized mouse plasmablast repertoire were assigned to the same cluster if the heavy chain V-gene, CDRH3 length, light chain V-gene, and CDRL3 length were identical. Sequences were further separated into putative lineages based on the degree of identity of the CDRH3 and CDRL3 sequences. Lineages were scored based on plasmablast cell frequency in the origin repertoire (score proportional to abundance), the degree of somatic hypermutation (SHM) in the complete heavy and light chain variable regions (score proportional to degree of SHM), and apparent convergent selection across animals (higher score for similar lineages observed in >1 mouse plasmablast repertoire that would have been considered the same lineage if the sequences originated in the same mouse). One, two, or three paired heavy and light chain sequences were selected for expression and screening from the lineages that received the highest scores.

### Vector construction and recombinant monoclonal antibody expression

DNA sequences for paired heavy chain (HC) and light chain (LC) IgG variable regions obtained through IRC technology (above) were synthesized and subcloned into expression vector pLEV123 (LakePharma, Inc.); HC variable region sequences were fused to the signal peptide MDPKGSLSWRILLFLSLAFELSYG, and human IgG1 constant regions. The LC variable region sequences were fused to the signal peptide MSVPTQVLGLLLLWLTDARC for the lambda LC, or METDTLLLWVLLLWVPGSTG for the kappa LC followed by the compatible human kappa or lambda LC constant regions.

HEK293 cells (ATCC) were seeded in shake flasks one day before transfection and grown using serum-free chemically defined media. The DNA expression plasmids were scaled up and transiently transfected into 10–30 ml of suspension HEK293 cells using LakePharma’s standard operating procedure for transient transfection. After 20 hours, cultures were fed and production continued for 5 days. Cells were sampled to obtain the viabilities and viable cell counts, and titers were measured (Octet QKe, ForteBio). On day 5, cells were sampled to obtain the viabilities and viable cell counts, and titers were measured (Octet QKe, ForteBio) before harvesting the cell cultures.

The conditioned media from HEK293 cells expressing antibody were harvested from the transient transfection production run by centrifugation. The supernatant was run over a Protein A column and eluted with a low pH buffer. Filtration using a 0.2 μm membrane filter was performed before aliquoting. After purification and filtration, the protein concentration was calculated from the OD280 and the extinction coefficient. Antibodies were formulated in HEPES buffer (200 mM HEPES-KOH, 100 mM NaCl, 50 mM NaOAc, pH 7.) CE-SDS analysis was performed (LabChip GXII, Perkin Elmer) to ensure antibody quality.

### Purification of recombinant proteins and preparation of complexes

Fabs and recombinant-shortened CSP (rsCSP) were produced as described previously [14, 34]. Samples for negative-stain EM (nsEM) were prepared by incubating Fabs and rsCSP overnight at a 10:1 molar ratio at 4° C and performing size-exclusion chromatography (GE Healthcare Superdex 200 16/60) the following day in Tris Buffered Saline (TBS: 50 mM Tris-HCl pH 8.0, 137 mM NaCl, 3.6 mM KCl). Samples for crystallography were prepared by mixing purified Fab in TBS at 11 mg/mL with peptide at a 1:5 molar ratio and incubating overnight at 4° C.

### Affinity measurements

Junctional versus NANP binding was determined using Biolayer Interferometry (BLI, Octet Red, Pall ForteBio). Biotinylated peptides, Biotin-linker-KQPADGNPDPNANPNV-NH_2_ (Junc) and Biotin-linker-NPNANPNANPNANPNA-NH_2_ (NPNA)_4_, were ordered from Innopep Inc. Peptides were diluted to 10 μg/mL in Kinetics buffer (Dulbecco’s PBS containing 0.002% Tween20 and 0.01% BSA) and captured on streptavidin sensors (Pall ForteBio, cat No 18–5019). The loaded sensors were dipped into solutions containing serial dilutions of Fab in Kinetics buffer (Fab317-(NPNA)_4_: 125 nM, 250 nM, 500 nM, 1000 nM; Fab317-Junc: 2000 nM, 4000 nM, 8000 nM; Fab667-(NPNA)_4_: 125 nM, 250 nM, 500 nM, 1000 nM; Fab667-Junc: 2000 nM, 4000 nM, 8000 nM; Fab668-(NPNA)_4_: 125 nM, 250 nM, 500 nM, 1000 nM; Fab668-Junc: 125 nM, 250 nM, 500 nM, 1000 nM). Fresh streptavidin sensors were used for each peptide-antibody interaction. In order to assess non-specific binding, each experiment included negative controls, in which unloaded sensors were dipped into antibody solution. Octet assays were carried out at 25⁰C and the data analyzed using the Octet Red Data Analysis software version 9.0.

Alanine scans were performed in BLI experiments (Octet QK384, Pall ForteBio) to determine the binding ability of mAbs 667 and 668 to CSP peptides ordered from Cambridge Peptides Ltd. A set of peptides was designed as an alanine scan for the DGNPDPNANPNV sequence. CSP peptides were diluted to 20 μg/mL in running buffer (1X HBS-EP + Buffer: Technova, cat. No. H8022). The biotinylated peptides were captured on the streptavidin sensors (Pall ForteBio, cat No 18–5019) and then the sensors were dipped into the antibody solutions at 30 μg/mL. Binding signals were double referenced using a well reference and a sensor reference. Data were analyzed using the Octet QK384 Data Analysis software version 8.2. A limit of detection of binding was determined specifically for each mAb and was used to differentiate non-binding and binding events for each peptide-antibody interaction.

### X-ray crystallography

The Ac-NPNANPNANPNA-NH_2_ [(NPNA)_3_] and Ac-KQPADGNPDPNANP-NH_2_ (Junc) peptides were ordered from Innopep Inc. at 98% purity. The Fab667-(NPNA)_3_ and Fab668-Junc complexes were crystallized from solutions containing Fab667 or Fab668 at 11 mg/mL in TBS buffer with a 5:1 molar ratio of peptide to Fab. All crystallization trials were performed using our high-throughput CrystalMation system (Rigaku, Carlsbad, CA) at TSRI. Crystals were grown using sitting-drop vapor diffusion (200 μl drop size) with a well solution containing 20% glycerol and 24% PEG1500 for the Fab667-(NPNA)_3_ complex, and 0.1 M HEPES-KOH (pH 6.96), 8% ethylene glycol, 16% PEG10000 for the Fab668-Junc complex. Crystals were grown at 293 K and typically appeared within 6 days. Fab667-(NPNA)_3_ crystals were cryo-cooled without additional cryoprotection, while Fab668-Junc crystals were cryoprotected by soaking in a well solution supplemented with 20% ethylene glycol. X-ray diffraction data were collected at SSRL BL12-2. S3 Table summarizes the data collection and processing statistics. The diffraction data were indexed, integrated, and scaled using the HKL-2000 package [35]. Since Fab668-Junc crystallized in space group P1, we merged two 360° datasets collected from different positions on the same crystal to improve the multiplicity of the data. The structures were solved by molecular replacement using PHASER [36] with a homology model [PIGSPro [37]] for either Fab667 or Fab668 as a search model. After refinement of the Fab using phenix.refine [38] combined with additional manual building cycles in Coot [39], clear Fo-Fc density was observed in the Fab-combining site for the peptide. The peptide was manually built into the difference density Fo-Fc map, followed by additional rounds of refinement of the complex in phenix.refine [38] using TLS and manual building cycles in Coot [39]. BSAs were calculated with the program MS [40] using a 1.7-Å) probe radius and standard van der Waals radii. Structures were validated using MolProbity [41].

### Molecular dynamics simulation

Fab-peptide complexes for the simulation were prepared from the crystal structure of Fab668-GNPDPNANP. The Gly in the peptide was removed to result in an 8-mer peptide and the termini were capped with N-terminal acetyl (ACE) and C-terminal N-methyl amide (NME) groups. The 8-mer peptide was mutated *in silico* to obtain NANPNANP, NVDPNANP, NANPNVDP, and PADGNPDP complexes with Fab668. The models were processed in Maestro (Schrodinger) where the Protein Preparation Wizard was used to build missing side-chains and model charge states of ionizable residues at neutral pH. An additional complex with protonated Asp^7^ of NANPNVDP was also prepared. Hydrogens and counter ions were added and the models were solvated in a cubic box of TIP4P-EW water [42] and 150mM KCl with a 10 Å buffer in AMBERtools [43].

AMBER16 [43] was used for energy minimization, heating, and equilibration steps, using the CPU code for minimization and heating and GPU code for equilibration. Systems were minimized by 1000 steps of hydrogen-only minimization, 2000 steps of solvent minimization, 2000 steps of ligand minimization, 2000 steps of side-chain minimization, and 5000 steps of all-atom minimization. Systems were heated from 0 K to 300 K linearly over 200 ps with 2 fs time-steps and 10.0 kcal/mol/Å position restraints on Fab and peptide. Temperature was maintained by the Langevin thermostat. Constant pressure equilibration with an 8 Å non-bonded cut-off was performed with 300 ps of protein and peptide restraints followed by 500ps without restraints in triplicate. A 10 Å cut-off for non-bonded interactions with particle mesh Ewald was used for a final 10 ns of equilibration for 10 independent runs of each triplicate. Production simulations were performed for 30 replicates of each complex on GPU enabled AMBER16 for 40 ns each (1.2 μs aggregate).

The energies of peptide binding to Fab were estimated by the molecular mechanics / generalized Born solvent accessibility (MM/GBSA) approach [44]. Energies were averaged over 3000 frames at 10 ps intervals aggregated from the last 1ns of 30 independent simulations of each complex in explicit solvent. Relative free energy differences were calculated from the molecular mechanical contributions between Fab and peptide and solvation energy components from generalized Born implicit solvent models. Physiological salt concentration of 0.15 M was used in the generalized Born model. Entropic contributions were not considered due to their large error and expected similarity between peptide variants. Energy calculations and decompositions were performed within Ambertools [43, 44].

For the simulation with the NANPNVDP peptide, constant pH replica exchange molecular dynamics (CpH-REMD) simulations in explicit solvent were run using CPU code implemented in AMBER16 [45]. Asp^7^ in NVDP and Asp^99^ in CDR H3, which are in proximity to each other and have the highest estimated pKa’s of Asp in the equilibrated Fab668-NANPNVDP complex, 4.8 and 4.9 respectively from PROPKA [46] as well as all Histidines, were allowed to be titratable during the simulation. Simulations commenced from an NVDP conformation with protonated Asp^7^ similar to the bound NANP conformation taken from 3 of the 30 independent runs of conventional MD with Asp^7^ constantly protonated, as described above. Six independent cpH-REMD simulations were performed with NVT at 300 K using the Langevin thermostat and a collision frequency of 5.0 ps^-1^. Simulations were run until protonated fractions converged, which occurred within 50 ns, and an additional 25 ns were performed to calculate pKa and the distribution of NVDP conformations. In this method, explicit solvent MD was run for 400 fs between protonation state change attempts using Generalized Born implicit solvent at 0.15 M salt. Metropolis Monte Carlo criteria, incorporating the simulation’s pH and the calculated free energy of changing protonation state, was used for acceptance of the protonation state change. Solvent was relaxed for 200 fs after a successful titration. Following relaxation, replica exchange along the pH coordinate was attempted to enhance sampling. Ten total constant pH simulations between pH 1 and 10 were run for each independent replica exchange set. Cphstats in AMBER16 was used to reconstruct trajectories for each pH and to calculate protonation fractions. Cpptraj in AMBER16 [43] was used to determine the Asp^7^ pairwise distances to the Fab668 pocket and in-house Python scripts were used to calculate populations on the Asp^7^-Fab668 distance coordinates.

### Negative stain electron microscopy

The rsCSP-Fab667 and rsCSP-Fab668 complexes were diluted to 0.01mg/ml using 1X TBS pH 7.4 and deposited on glow discharged carbon-coated copper-mesh grids. 2% uranyl formate was used to negatively stain the complexes for 50 seconds. Grids were transferred to a Thermo Fisher Tecnai Spirit T12 (120 keV) equipped with a TVIPS CMOS 4k x 4k camera for data collection. The Leginon software [47] was used for automated data collection and resulting images were stored in the Appion database [48]. All images were collected at 52,000 x magnification, a defocus value of -1.5 um, a dose of 25e^-^/Å^2^, and a pixel size of 2.05.

A tilted dataset was collected for rsCSP-Fab667 and rsCSP-Fab668 complexes by changing the stage angle to 0°, -10°, -20°, -30°, -40°, and -50°, resulting in 251 and 505 images, respectively. Particles were picked using DogPicker [49] and stacked with a box size of 160 or 192 pixels. Micrographs were CTF corrected using GCTF [50] and particles were extracted. The particle stack was imported into cryoSparc2 [51], where rounds of reference-free 2D classifications, initial model-free 3D classifications and 3D refinements were performed. 3D refinements did not have symmetry imposed. Crystal structures of Fab667 and 668 were fit to their respective nsEM maps using UCSF Chimera [52].

### Field flow fractionation coupled to multi-angle light scattering

50ug of each previously SEC purified rsCSP-Fab667 and rsCSP-Fab668 complexes were loaded onto a short channel fast field fractionation (FFF) unit on the Eclipse Dualtec system (Wyatt Technologies). This unit was paired to an Agilent 1260 Infinity II Liquid Chromatography system (Agilent Technologies) with a MiniDawn Treos multi-angle light scattering detector and an Optilab T-reX refractive index (RI) detector (Wyatt Technologies). The molecular masses and radii of hydration of the two complexes were calculated in the ASTRA V software (Wyatt Technologies) with the resulting UV_280_, light scattering, and refractive index data. The value for the change in refractive index over change in concentration (dn/dc) was 0.185.

### In vivo assessment of liver burden load

Transgenic *P*. *berghei* sporozoites that now express *P falciparum* CSP on their surface were produced in *A*. *stephensi* mosquitos that fed on parasite-infected mice. Between days 20–23, after feeding, the salivary glands of mosquitoes were dissected and disrupted in HBSS medium and sporozoites were isolated and counted in a hemocytometer. Six- to eight-week-old female C57Bl/6 mice were purchased from Charles River Laboratories (Frederick, MD) and maintained in the animal facility at the Johns Hopkins University, Bloomberg School of Public Health. Mice were injected IV with the indicated immunoglobulin in 200 μl and 2h later challenged with 2000 transgenic *P*. *berghei* sporozoites expressing *P falciparum* CSP and GFP-Luc. After 42 h, mice were injected with 100 μl of D-luciferin (30 mg/ml), anesthetized with isoflurane, and the parasite liver load was measured by bioluminescence on an IVIS Spectrum imager. The results were expressed as total flux (photons/second). Fold reduction was measured as the ratio of the total flux in naïve infected mice versus mice with passive transferred antibodies.

### Ethics statement

The liver burden assay was carried out in strict accordance with the recommendations in the Guide for the Care and Use of Laboratory Animals of the National Institutes of Health. The protocol was approved by the Animal Care and Use Committee of the Johns Hopkins University (protocol number MO18H419).

The immunizations of Kymab mice in this study were carried out under a Project License (PPL80/2432) issued by the UK Government Home Office under Animal (Scientific Procedures) Act (A(SP)A), 1986, incorporating Directive 2010/63/EU of the European Parliament and with the approval of the Sanger Institute Animal Welfare and Ethical Review Body. The Institute complied with the Code of Practice issued by the UK Government which complies with the A(SP)A. The Institute has a PHS assurance F16-00128 (WTSI).

## Supporting information

S1 Table Summary of seven Kymab-Atreca anti-CSP mAbs: in vitro and in vivo assays. (DOCX)

S2 Table Affinity measurements. Binding of Fabs with selected peptides using Biolayer Interferometry. Binding data of the junctional peptide with Fab667 were fitted to a biphasic binding model. Reported errors are fitting errors.
(DOCX)

S3 Table Data collection and refinement statistics for Fab667-(NPNA)_3_ and Fab668-Junc crystal structures. (DOCX)

S4 Table Statistical analysis of the in vivo protection data. (DOCX)

S1 Fig Antibody lineage selection data. A total of 13 IgG sequence repertoires were generated by individual sequencing of 2588 plasmablasts from 7 HK Kymice and 6 HL Kymice (108–343 heavy and light chain pairs per mouse). A total of 1321 antibody lineages were detected with these sequence repertoires (see Methods). Lineages were binned according to the number of plasmablasts in each lineage expressed as a percent of the total plasmablasts sequenced from the relevant animal (percent of repertoire). Among these lineages, 45 were selected (green) and 1–3 specific antibody sequences were chosen per selected lineage for recombinant expression (see Methods). For the selection criterion regarding apparent convergence across animals, lineages that showed convergent features with selected lineages, but were not themselves selected, are shown in blue. Most of the largest percentage lineages were chosen for expression and testing with some smaller lineages chosen mostly based on apparent convergent features with lineages in other animals.
(TIF)

S2 Fig Electron density and conformation of the bound peptide. 2Fo-Fc (blue) and composite omit 2Fo-Fc (green) electron density map for the peptide (yellow carbons) bound to (**A**) Fab667 and (**B**) Fab668. The 2Fo-Fc density is contoured at 1.6σ (dark blue) and 0.8σ (cyan), while the omit 2Fo-Fc density is contoured at 0.8σ. The Fab is shown in cartoon representation with the heavy and light chains colored black and white respectively. (**C**) Overlay of the 667-bound peptide (cyan carbons and labels) and the 668-bound peptide (yellow carbons, orange labels). The peptides align well around the centrally located NANP repeat, which is numbered in the figure. The NAN sequence of this repeat adopts an Asn pseudo 3_10_ turn as observed previously for NPN sequences.
(TIF)

S3 Fig Buried surface area (BSA) analysis of the bound peptide on Fab 667 and Fab 668. The BSA (Å^2^) for each amino acid (main-chain + side-chain) for the peptides bound to (**A**) Fab667 and (**B**) Fab 668. Residues are numbered using the same numbering scheme as in Fig 3 and S1 Fig.
(TIF)

S4 Fig Interactions of NANP in the Junc and (NPNA)_3_ peptides with Fab668 and Fab667. (**A**) Hydrogen bond network for Asn11 and Asn13 of the junctional peptide with Fab668. The CDR loops are colored red for H3, blue for H2 and green for H1. The peptide is shown in stick configuration (yellow carbons). Hydrogen bonds are shown as black dashes. (**B**) Hydrophobic interactions for Ala12 of the same peptide with Fab668. Distances are shown as black dashes and are labeled. CDR L3 is colored pink. (**C**) Hydrogen bond network for Asn3 and Asn5 of the NANP peptide with Fab667. The CDR loops are colored red for H3, blue for H2 and green for H1. The peptide is shown in stick configuration (yellow carbons). Hydrogen bonds are shown as black dashes. (**D**) Hydrophobic interactions for Ala4 of the same peptide with Fab667. CH/π interactions and distances are shown as black dashes and labeled. CDR L3 is colored pink.
(TIF)

S5 Fig Protonation-dependent conformations of Asp^7^ (in NANPNVDP) and Asn^7^ (in NANPNANP). (**A**) Deprotonated (charged) Asp^7^ samples an out-of-pocket orientation. The deprotonated (charged) Asp^7^ side-chain conformation samples a wide distribution as represented by the distance between Asp^7^ Oδ2 and Gly^96^ O as well as between Asp^7^ Cγ and Asn^52^ Cγ. Snapshots of the deprotonated Asp^7^ simulation are shown. (**B**) Asn^7^ favors an in-pocket conformation as illustrated by its distribution along the Asn^7^ Nδ2 –Gly^96^ O distance coordinate. Representative frames of the protonated Asn^7^ simulation are shown. (**C**) Protonated (uncharged) Asp^7^ also prefers an in-pocket conformation.
(TIF)

S6 Fig pKa of Asp^7^ (in NANPNVDP) from constant pH replica exchange MD. Asp^7^ pH titration curve from ensemble averaged protonation fractions for 10 sampled pH values over which replica exchange was employed. Six independent sets of replica exchange over 10 constant pH simulations of 25ns each were used. The titration curve can be fit with a pKa of 6.1 and a Hill coefficient of 0.98.
(TIF)

S7 Fig Negative-stain electron microscopy Fourier Shell Correlations. FSC curves are shown for (**A**) Fab667-rsCSP and (**B**) Fab668-rsCSP. The resolution for 3D reconstructions is 18 Å for Fab667-rsCSP and 17 Å for Fab668-rsCSP, as determined by the FSC 0.5 criterion.
(TIF)

S8 Fig Field flow fractionation coupled to multi-angle light scattering to approximate the molecular mass for selected rsCSP complexes. (**A**) Fab667-rsCSP and (**B**) Fab668-rsCSP complexes. The chromatogram is shown in black and the molecular mass estimation is shown in red. The shoulder on the right of the main peak was not used to approximate the reported molecular mass.
(TIF)
