# Supplementary material for: Structure and mechanism of monoclonal antibody binding to the junctional epitope of Plasmodium falciparum circumsporozoite protein
Source: PLoS Pathog. 2020 Mar 9;16(3):e1008373. doi: 10.1371/journal.ppat.1008373 (PMC7082059; doi:10.1371/journal.ppat.1008373)
Supplement: S1 Table — (DOCX) [file ppat.1008373.s001.docx]

**S1 Table.** Summary of seven Kymab-Atreca anti-CSP mAbs in in vitro and in vivo assays.

| **Antibody ID** | **ELISA** | **IFA** | **Epitope^1^** | **Protection (%)^2^** |
| --- | --- | --- | --- | --- |
| 640 | +++ | + | Cterm | 0 |
| 643 | +++ | + (faint) | Cterm | 0 |
| 646 | ++ | + | Cterm | 0 |
| 649 | +++ | + | ND^3^ | 0 |
| 662 | +++ | + | Cterm | 35 |
| 667 | +++ | + | NANP/Junc^4^ | 100 |
| 668 | +++ | + | NANP/Junc | 100 |

^1^Primary binding assays screening were to full-length CSP, N-terminal, C-terminal and NANP repeat regions, and subsequently expanded to junctional peptide binding analysis for 667 & 668.

^2^Liver burden assay (300μg/mouse, 6 mice per antibody).

^3^Not determined- binding to full-length CSP was positive, but no binding was detected to either the Cterm (αTSR domain) or NANP peptides.

^4^During *in vitro* & *in vivo* functional characterization, further peptide binding studies showed 667 & 668 bound the junctional peptide.
