# Supplementary material for: Structure and mechanism of monoclonal antibody binding to the junctional epitope of Plasmodium falciparum circumsporozoite protein
Source: PLoS Pathog. 2020 Mar 9;16(3):e1008373. doi: 10.1371/journal.ppat.1008373 (PMC7082059; doi:10.1371/journal.ppat.1008373)
Supplement: S2 Table — Binding of Fabs with selected peptides using Biolayer Interferometry. Binding data of the junctional peptide with Fab667 were fitted to a biphasic binding model. Reported errors are fitting errors. (DOCX) [file ppat.1008373.s002.docx]

**S2 Table.** Binding of Fabs with selected peptides using Biolayer Interferometry. Binding data of the junctional peptide with Fab667 were fitted to a biphasic binding model.

|  | K_d_  (nM) | k_on_  (x10^4^ M^-1^s^-1^) | k_off_  (x10^-3^ s^-1^) | K_d2_  (nM) | k_on2_  (x10^4^ M^-1^s^-1^) | k_off2_  (x10^-3^ s^-1^) |
| --- | --- | --- | --- | --- | --- | --- |
| Fab317-NANP | 25.0 ± 0.3 | 5.84 ± 0.03 | 1.46 ± 0.02 |  |  |  |
| Fab667-NANP | 176 ± 1 | 2.44 ± 0.01 | 4.29 ± 0.02 |  |  |  |
| Fab668-NANP | 55.6 ± 0.5 | 3.22 ± 0.02 | 1.79 ± 0.01 |  |  |  |
| Fab317-Junc | NB^1^ | NB | NB |  |  |  |
| Fab667-Junc | 12000 ± 1000 | 0.028 ± 0.003 | 3.47 ± 0.04 | 4600 ± 300 | 12.7 ± 0.7 | 580 ± 10 |
| Fab668-Junc | 206 ± 1 | 2.065 ± 0.009 | 4.24 ± 0.01 |  |  |  |

^1^ Non-Binding
