# Supplementary material for: Structure and mechanism of monoclonal antibody binding to the junctional epitope of Plasmodium falciparum circumsporozoite protein
Source: PLoS Pathog. 2020 Mar 9;16(3):e1008373. doi: 10.1371/journal.ppat.1008373 (PMC7082059; doi:10.1371/journal.ppat.1008373)
Supplement: S3 Table — (DOCX) [file ppat.1008373.s003.docx]

**S3 Table.** Data collection and refinement statistics for Fab667-(NPNA)_3_ and Fab668-Junc crystal structures.

| Data collection | Fab668-Junc | Fab667-(NPNA)_3_ |
| --- | --- | --- |
| Beamline | SSRL12-2 | SSRL12-2 |
| Wavelength (Å) | 0.97946 | 0.97946 |
| Space group | P1 | P3_2_21 |
| Unit cell parameters (Å, °) | a=58.07, b=60.13, c=76.34 | a=b=70.40, c=186.49 |
|  | α=72.43, β=68.07, γ=83.58 |  |
| Resolution (Å) | 50.00-1.57 (1.60-1.57)^a^ | 50.00-2.05 (2.09-2.05)^a^ |
| Unique Reflections | 111,126 (5,151)^a^ | 32,790 (1,304)^a^ |
| Multiplicity | 7.0 (4.6)^a^ | 9.8 (3.2)^a^ |
| Completeness (%) | 86.8 (80.7)^a^ | 97.7 (78.8)^a^ |
| <I/σ_I_> | 23.3 (2.2)^a^ | 12.0 (1.1)^a^ |
| R_sym_^b^ (%) | 18.1 (77.3)^a^ | 17.1 (75.9)^a^ |
| R_pim_^b^ (%) | 7.2 (38.9)^a^ | 5.4 (43.2)^a^ |
| CC_1/2_^c^ (%) | 90.7 (70.3)^a^ | 90.7 (70.6)^a^ |
| Refinement statistics |  |  |
| Resolution (Å) | 35.05-1.57 | 43.53-2.06 |
| Reflections (work) | 105,599 | 31,104 |
| Reflections (test) | 5483 | 1605 |
| R_cryst_^d^ / R_free_^e^ (%) | 17.7/21.2 | 20.2/25.8 |
| No. of atoms |  |  |
| Protein | 6741 | 3320 |
| Water | 778 | 166 |
| Buffer | 8 | 16 |
| Average B-value (Å^2^) |  |  |
| Fab | 25 ± 9 | 53 ± 12 |
| Peptide | 27 ± 9 | 55 ± 9 |
| Water | 34 ± 9 | 55 ± 12 |
| Buffer | 43 ± 4 | 82 ± 12 |
| Wilson B-value (Å^2^) | 18 | 42 |
| RMSD from ideal geometry |  |  |
| Bond length (Å) | 0.014 | 0.002 |
| Bond angle (°) | 1.40 | 0.53 |
| Ramachandran statistics^f^ |  |  |
| Favored (%) | 97.92 | 98.14 |
| Outliers (%) | 0.00 | 0.23 |
| Clashscore | 3.75 | 1.83 |

^a^ Numbers in parentheses refer to the highest resolution shell.

^b^ *R*_sym_ = Σ*_hkl_* Σ*_i_* | I*_hkl,i_* - <I*_hkl_*> | / Σ*_hkl_* Σ*_i_* I*_hkl,i_* and R*_pim_* = Σ*_hkl_* (1/(n-1))^1/2^ Σ*_i_* | I*_hkl,i_* - <I*_hkl_*> | / Σ*_hkl_* Σ*_i_* I*_hkl,i_*, where I*_hkl,i_* is the scaled intensity of the i^th^ measurement of reflection h, k, l, <I*_hkl_*> is the average intensity for that reflection, and *n* is the redundancy.

^c^ CC_1/2_ = Pearson correlation coefficient between two random half datasets.

*^d^ R*_cryst_ = Σ*_hkl_* | *F*_o_ - *F*_c_ | / Σ*_hkl_* | *F*_o_ | x 100, where *F*_o_ and *F*_c_ are the observed and calculated structure factors, respectively.

^e^ *R*_free_ calculated as for *R*_cryst_, but on a test set comprising 5% of the data excluded from refinement.

^f^ From MolProbity (*41*).
