# Supplementary material for: Structure and mechanism of monoclonal antibody binding to the junctional epitope of Plasmodium falciparum circumsporozoite protein
Source: PLoS Pathog. 2020 Mar 9;16(3):e1008373. doi: 10.1371/journal.ppat.1008373 (PMC7082059; doi:10.1371/journal.ppat.1008373)
Supplement: S4 Table — (DOCX) [file ppat.1008373.s004.docx]

**S4 Table.** Statistical analysis of the in vivo protection data.

| **Mann-Whitney** | **AB317 vs AB-668** | | | |
| --- | --- | --- | --- | --- |
|  | 300 µg | 100 µg | 30 µg | 10 µg |
| P value | 0.0079 | 0.0079 | 0.0079 | 0.5317 |
| Exact or Approx. | exact | exact | exact | exact |
| P value summary | Significant** | Significant** | Significant** | ns^1^ |
| **Mann-Whitney** | **AB317 vs AB-667** | | | |
|  | 300 µg | 100 µg | 30 µg | 10 µg |
| P value | 0.0079 | 0.0079 | 0.0159 | 0.2222 |
| Exact or Approx. | exact | exact | exact | exact |
| P value summary | Significant** | Significant** | Significant* | ns |
| **Mann-Whitney** | **AB-667 vs AB-668** | | | |
|  | 300 µg | 100 µg | 30 µg | 10 µg |
| P value | 0.0079 | 0.0159 | 0.2222 | 0.0556 |
| Exact or Approx. | exact | exact | exact | exact |
| P value summary | Significant** | Significant* | ns | ns |

^1^not significant
