# Supplementary figures and images for: Structure and mechanism of monoclonal antibody binding to the junctional epitope of Plasmodium falciparum circumsporozoite protein
Source: PLoS Pathog. 2020 Mar 9;16(3):e1008373. doi: 10.1371/journal.ppat.1008373 (PMC7082059; doi:10.1371/journal.ppat.1008373)

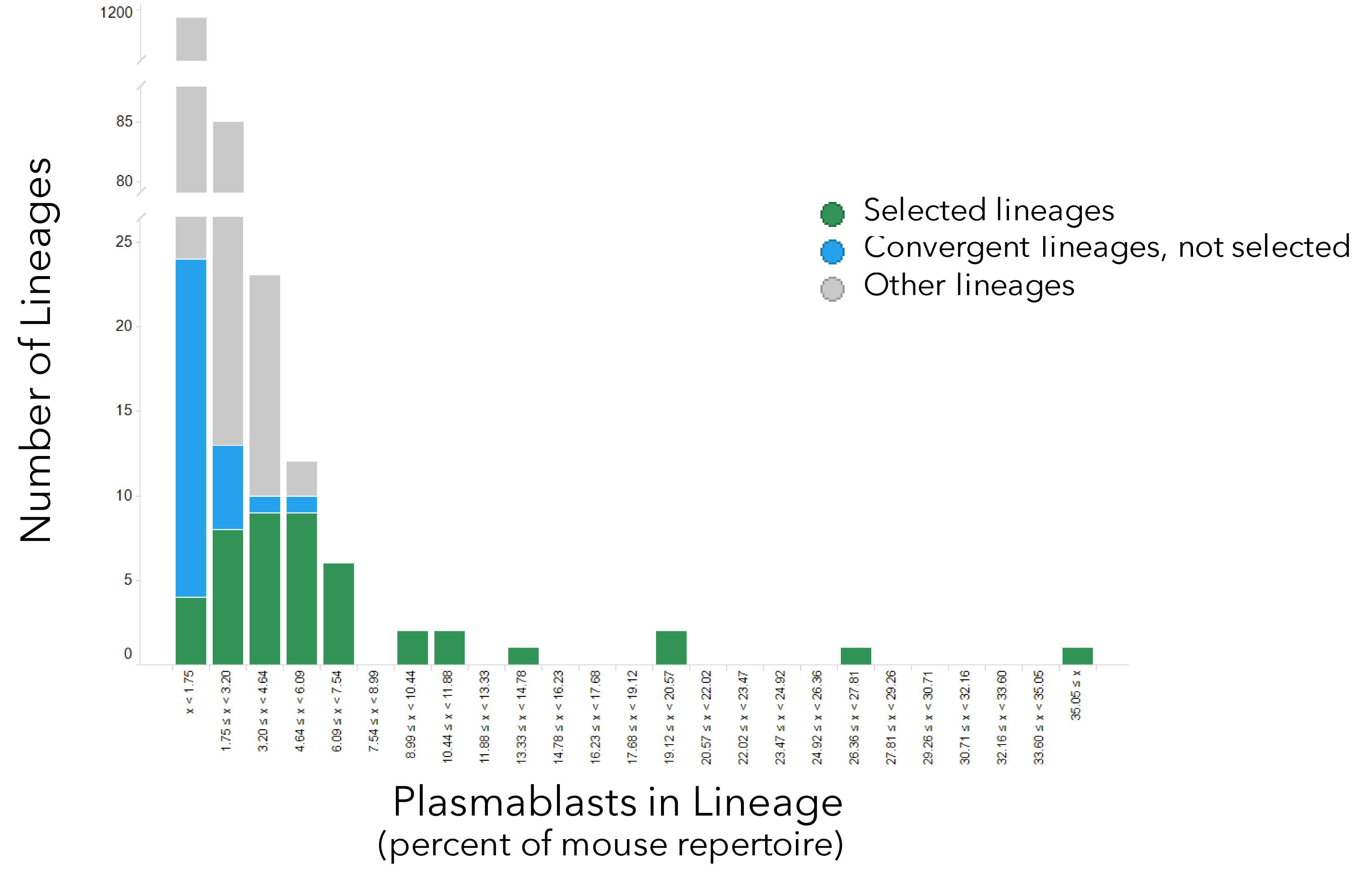

Supplement: S1 Fig — A total of 13 IgG sequence repertoires were generated by individual sequencing of 2588 plasmablasts from 7 HK Kymice and 6 HL Kymice (108–343 heavy and light chain pairs per mouse). A total of 1321 antibody lineages were detected with these sequence repertoires (see Methods). Lineages were binned according to the number of plasmablasts in each lineage expressed as a percent of the total plasmablasts sequenced from the relevant animal (percent of repertoire). Among these lineages, 45 were selected (green) and 1–3 specific antibody sequences were chosen per selected lineage for recombinant expression (see Methods). For the selection criterion regarding apparent convergence across animals, lineages that showed convergent features with selected lineages, but were not themselves selected, are shown in blue. Most of the largest percentage lineages were chosen for expression and testing with some smaller lineages chosen mostly based on apparent convergent features with lineages in other animals. (TIF) [file ppat.1008373.s005.tif]

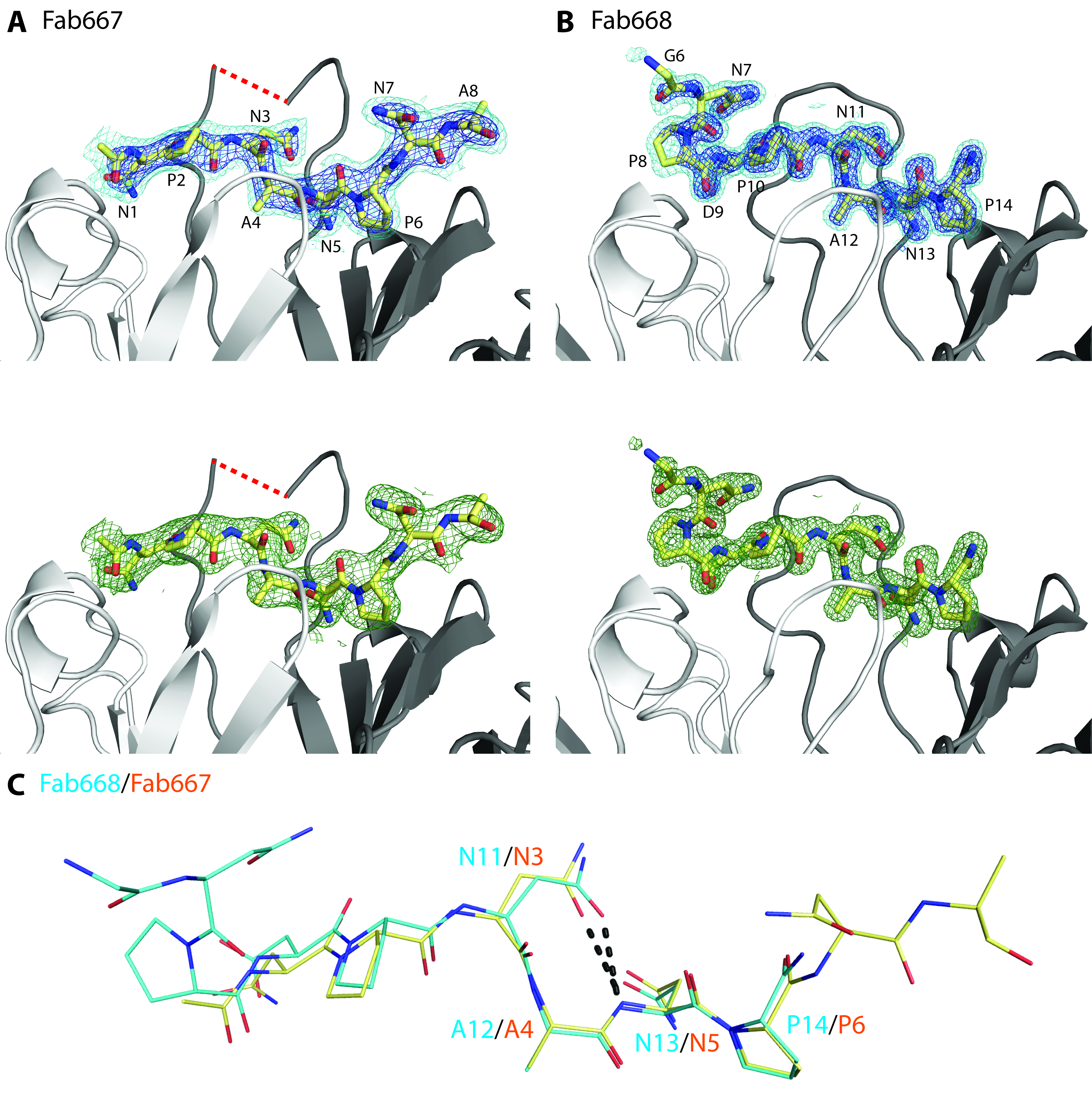

Supplement: S2 Fig — 2Fo-Fc (blue) and composite omit 2Fo-Fc (green) electron density map for the peptide (yellow carbons) bound to (A) Fab667 and (B) Fab668. The 2Fo-Fc density is contoured at 1.6σ (dark blue) and 0.8σ (cyan), while the omit 2Fo-Fc density is contoured at 0.8σ. The Fab is shown in cartoon representation with the heavy and light chains colored black and white respectively. (C) Overlay of the 667-bound peptide (cyan carbons and labels) and the 668-bound peptide (yellow carbons, orange labels). The peptides align well around the centrally located NANP repeat, which is numbered in the figure. The NAN sequence of this repeat adopts an Asn pseudo 310 turn as observed previously for NPN sequences. (TIF) [file ppat.1008373.s006.tif]

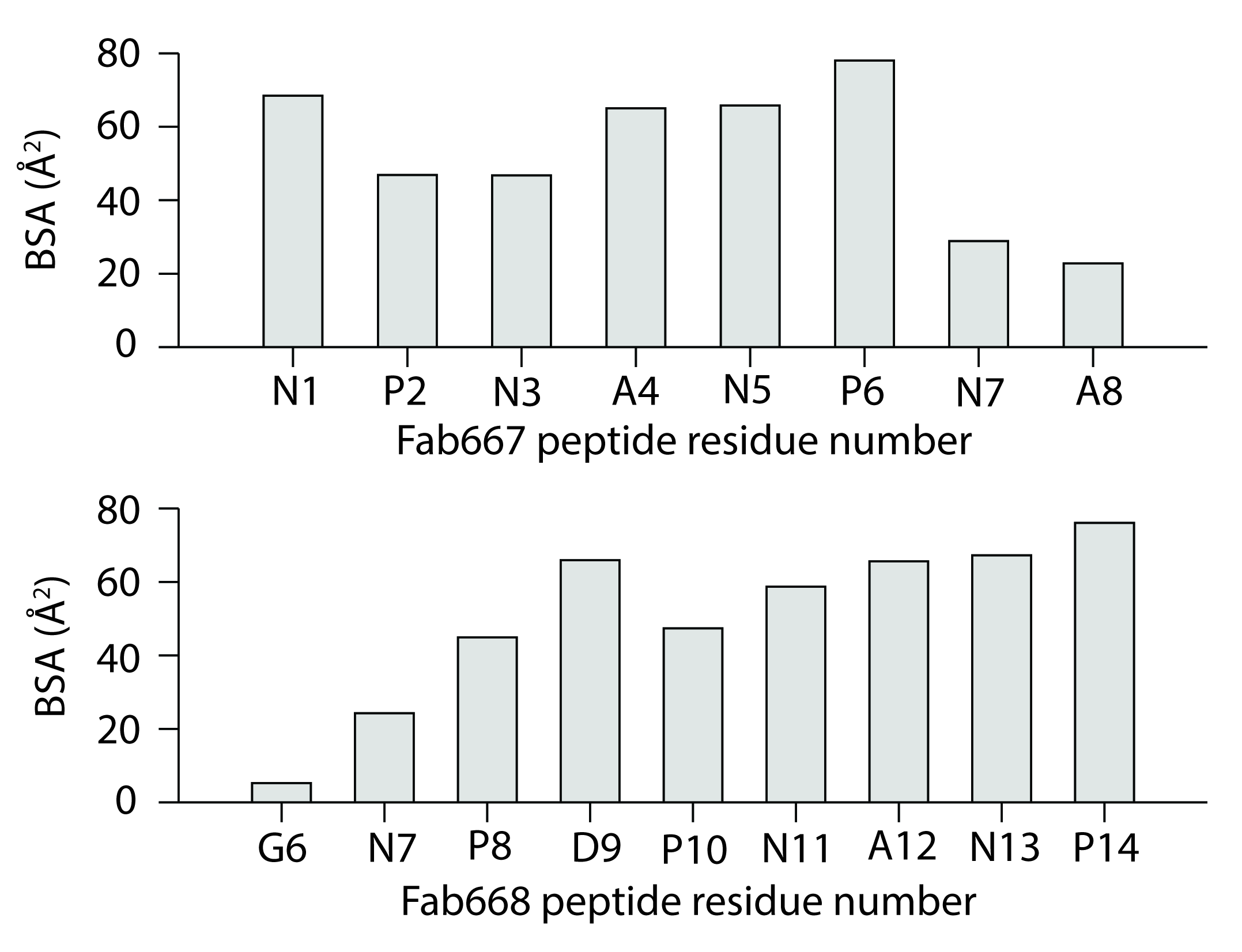

Supplement: S3 Fig — The BSA (Å2) for each amino acid (main-chain + side-chain) for the peptides bound to (A) Fab667 and (B) Fab 668. Residues are numbered using the same numbering scheme as in Fig 3 and S1 Fig. (TIF) [file ppat.1008373.s007.tif]

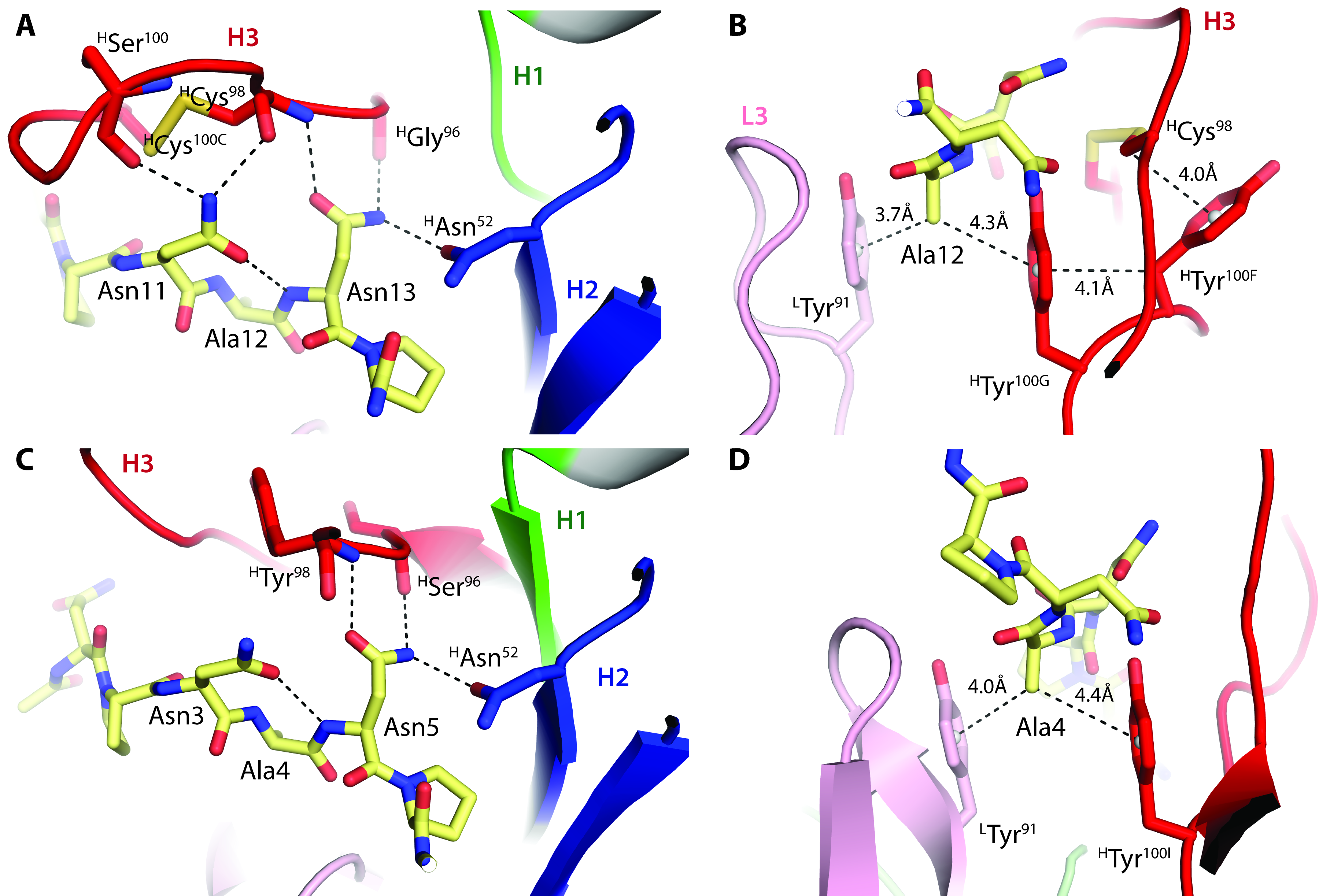

Supplement: S4 Fig — (A) Hydrogen bond network for Asn11 and Asn13 of the junctional peptide with Fab668. The CDR loops are colored red for H3, blue for H2 and green for H1. The peptide is shown in stick configuration (yellow carbons). Hydrogen bonds are shown as black dashes. (B) Hydrophobic interactions for Ala12 of the same peptide with Fab668. Distances are shown as black dashes and are labeled. CDR L3 is colored pink. (C) Hydrogen bond network for Asn3 and Asn5 of the NANP peptide with Fab667. The CDR loops are colored red for H3, blue for H2 and green for H1. The peptide is shown in stick configuration (yellow carbons). Hydrogen bonds are shown as black dashes. (D) Hydrophobic interactions for Ala4 of the same peptide with Fab667. CH/π interactions and distances are shown as black dashes and labeled. CDR L3 is colored pink. (TIF) [file ppat.1008373.s008.tif]

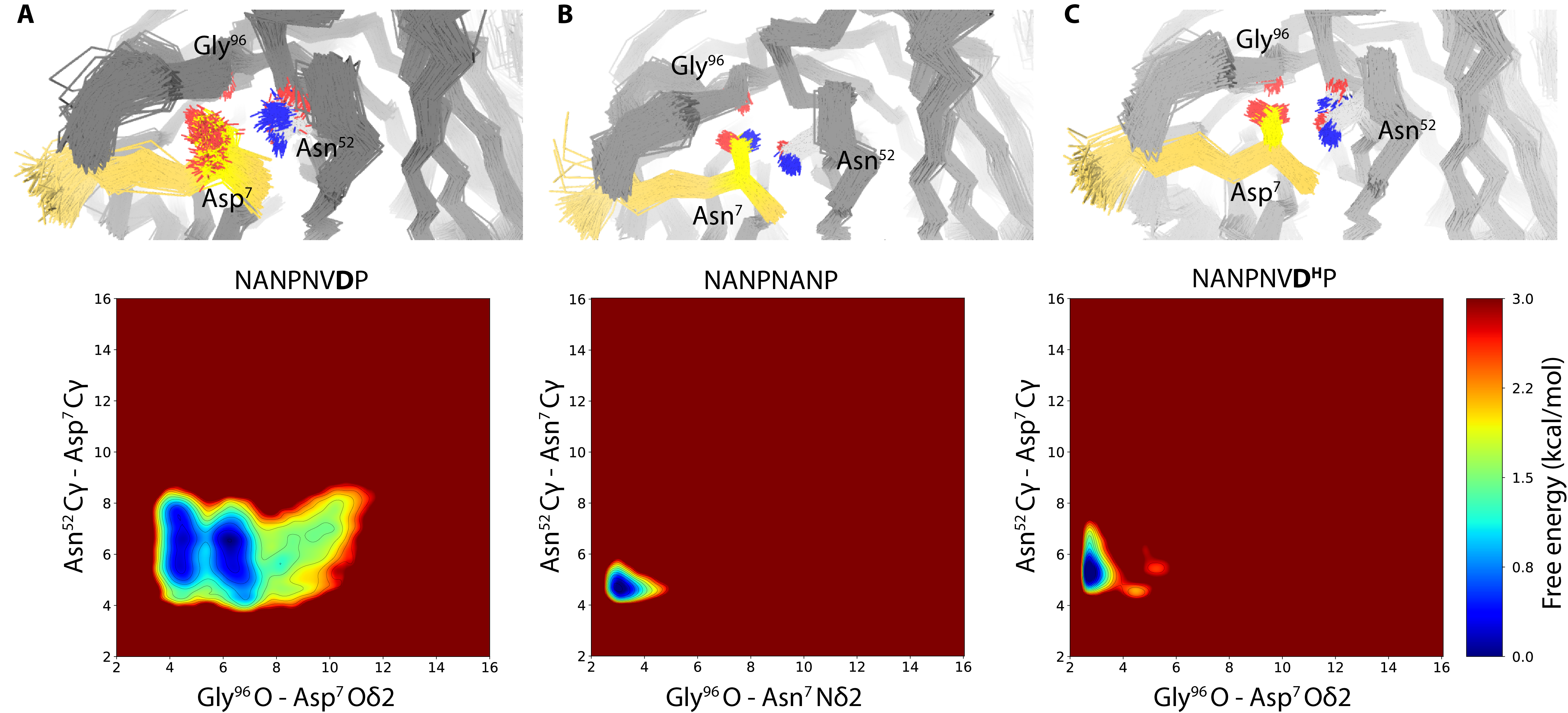

Supplement: S5 Fig — (A) Deprotonated (charged) Asp7 samples an out-of-pocket orientation. The deprotonated (charged) Asp7 side-chain conformation samples a wide distribution as represented by the distance between Asp7 Oδ2 and Gly96 O as well as between Asp7 Cγ and Asn52 Cγ. Snapshots of the deprotonated Asp7 simulation are shown. (B) Asn7 favors an in-pocket conformation as illustrated by its distribution along the Asn7 Nδ2 –Gly96 O distance coordinate. Representative frames of the protonated Asn7 simulation are shown. (C) Protonated (uncharged) Asp7 also prefers an in-pocket conformation. (TIF) [file ppat.1008373.s009.tif]

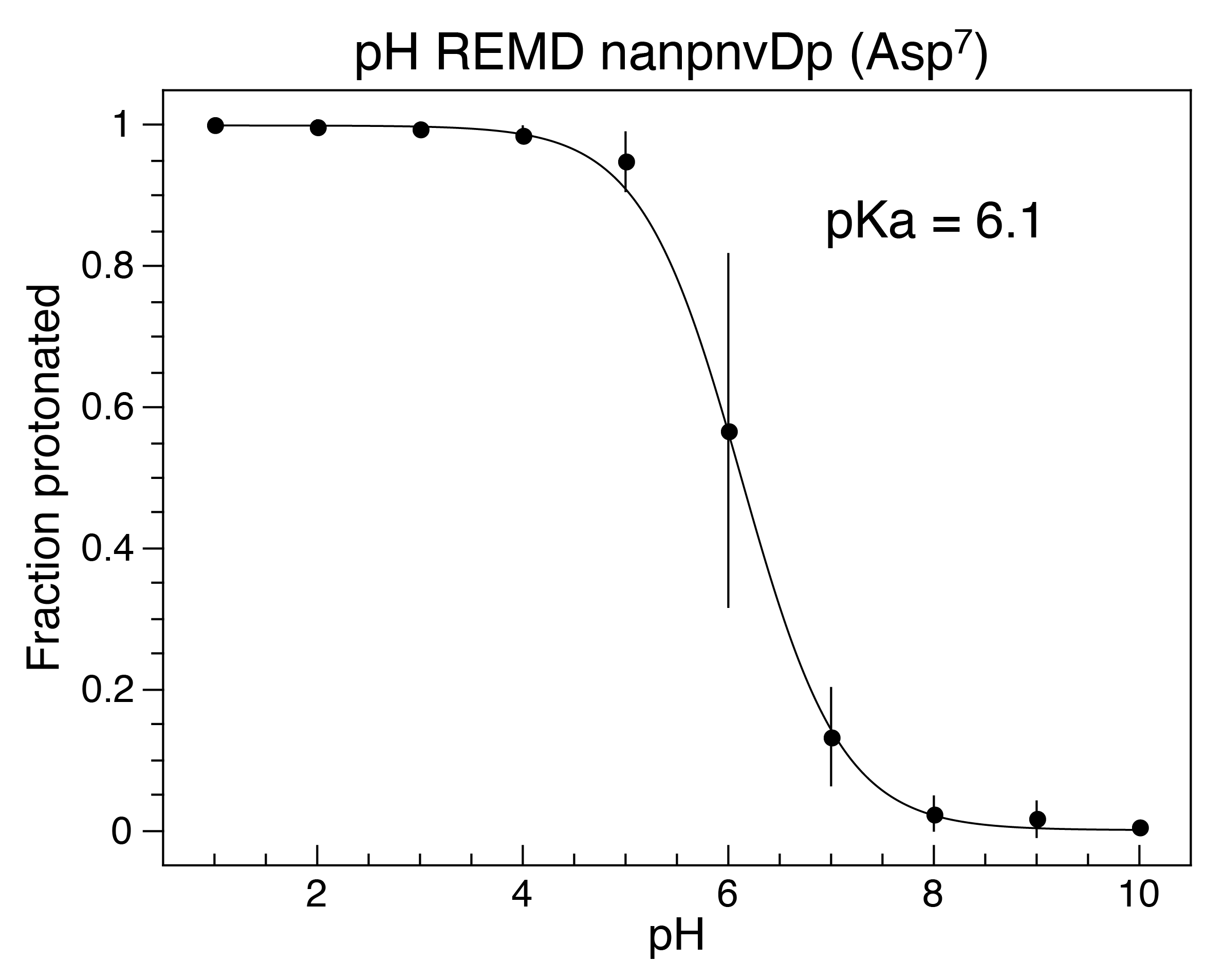

Supplement: S6 Fig — Asp7 pH titration curve from ensemble averaged protonation fractions for 10 sampled pH values over which replica exchange was employed. Six independent sets of replica exchange over 10 constant pH simulations of 25ns each were used. The titration curve can be fit with a pKa of 6.1 and a Hill coefficient of 0.98. (TIF) [file ppat.1008373.s010.tif]

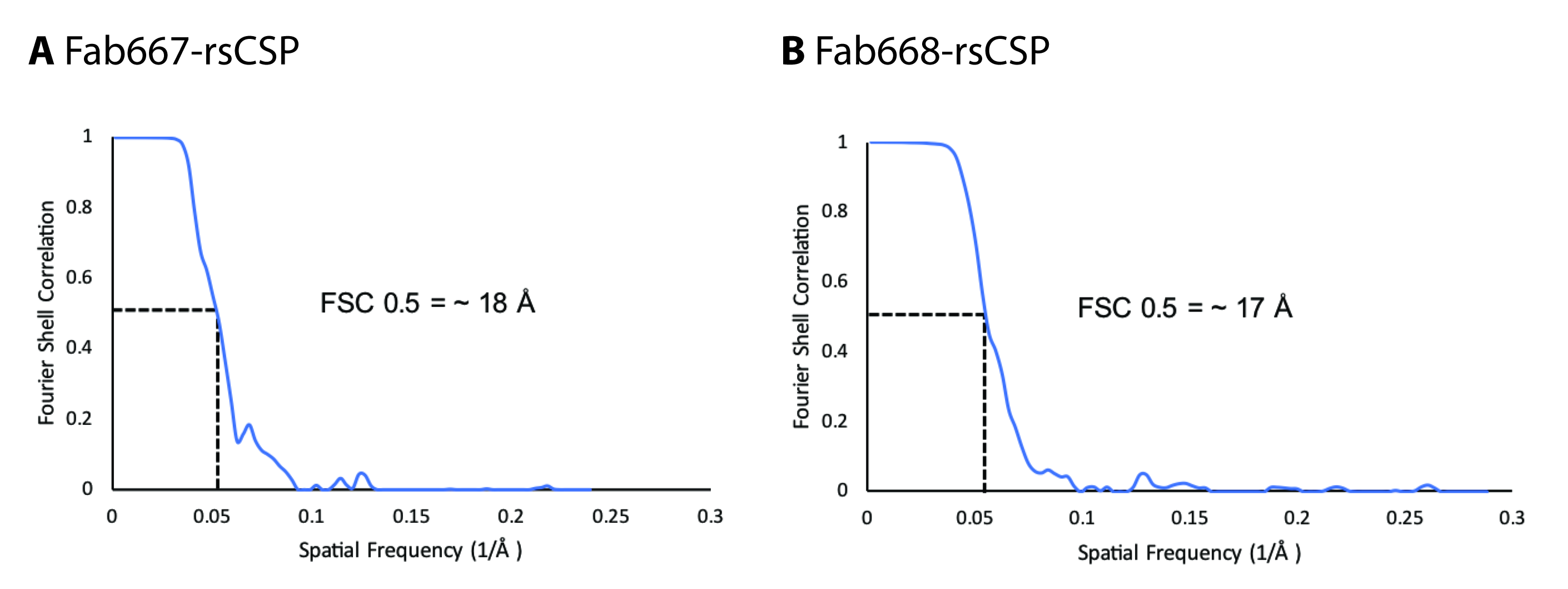

Supplement: S7 Fig — FSC curves are shown for (A) Fab667-rsCSP and (B) Fab668-rsCSP. The resolution for 3D reconstructions is 18 Å for Fab667-rsCSP and 17 Å for Fab668-rsCSP, as determined by the FSC 0.5 criterion. (TIF) [file ppat.1008373.s011.tif]

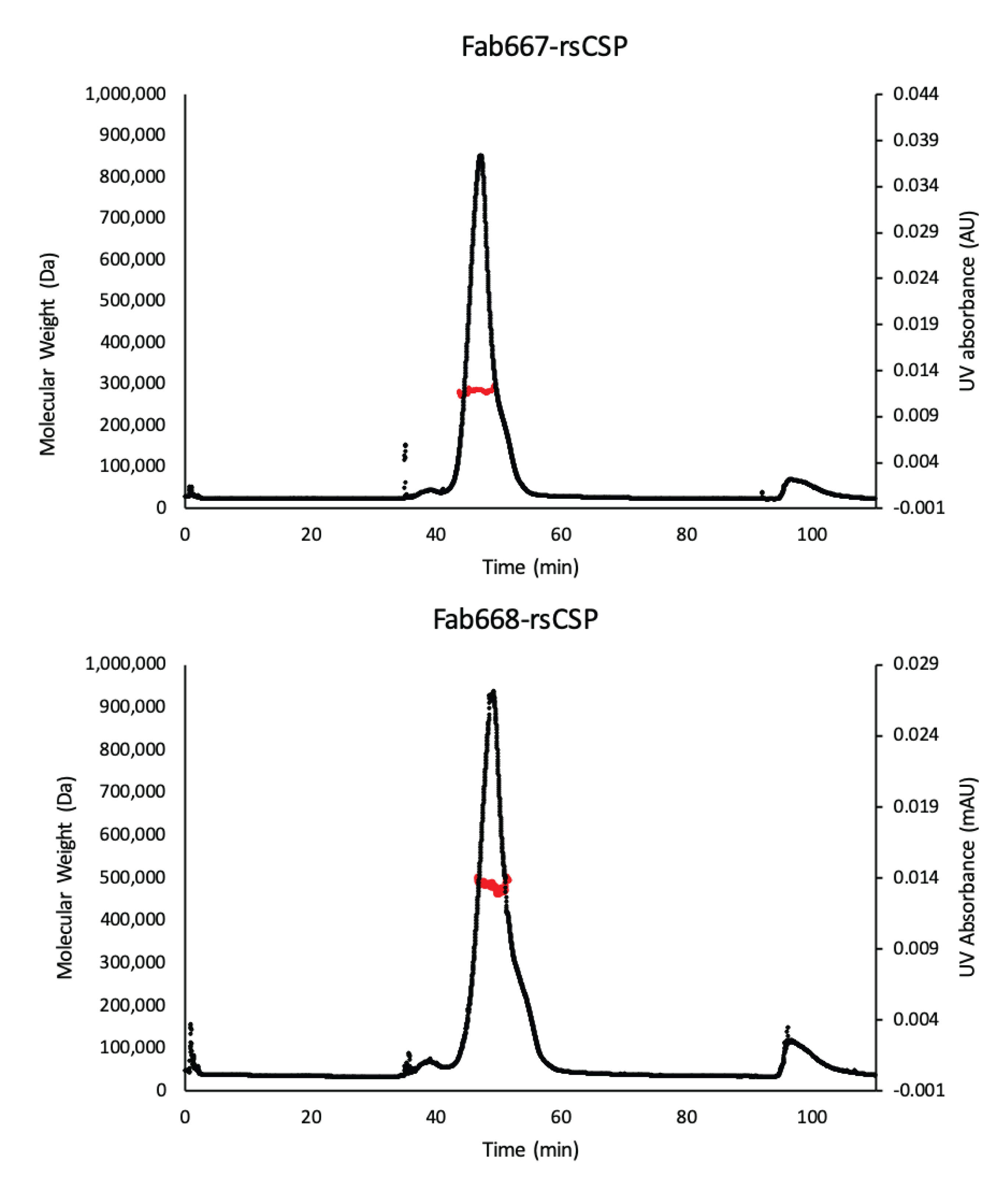

Supplement: S8 Fig — (A) Fab667-rsCSP and (B) Fab668-rsCSP complexes. The chromatogram is shown in black and the molecular mass estimation is shown in red. The shoulder on the right of the main peak was not used to approximate the reported molecular mass. (TIF) [file ppat.1008373.s012.tif]
